# Strong upregulation of inflammatory genes accompanies photoreceptor demise in canine models of retinal degeneration

**DOI:** 10.1371/journal.pone.0177224

**Published:** 2017-05-09

**Authors:** Tatyana Appelbaum, Evelyn Santana, Gustavo D. Aguirre

**Affiliations:** Section of Ophthalmology, School of Veterinary Medicine, University of Pennsylvania, Philadelphia, Pennsylvania, United States of America; University of Cologne, GERMANY

## Abstract

We have analyzed the complex pattern of the inflammatory response in early-onset canine models of human retinitis pigmentosa, rcd1, xlpra2 and erd, as well as late-onset xlpra1, in comparative manner. The time course of immune response genes and proteins expression was examined along the timeline of photoreceptors degeneration. Gene expression analysis of the early-onset models prior to and after the peak of photoreceptors death identified the involvement of multiple immune response genes including those encoding constituents of the NLRP3 inflammasome, its substrates, pro-IL1B, pro-IL18, and common components of IL1B, IL18 and TLR4 pathways. Out of two activated caspase-1 cleavage products, IL1B and IL18, only IL1B was detected in rcd1 and xlpra2 while precursor IL18 remained unprocessed in the same protein extract highlighting prominence of IL1B pathway. An overall immune response was most prominent in rcd1 followed by xlpra2 and least prominent in erd. Noticeably, in rcd1 and xlpra2, but not in erd, early induction of the immune response was accompanied by sustained intraretinal migration and activation of retinal microglia. Lastly, delayed activation of the anti-inflammatory factors in all early-onset models was insufficient to counterbalance rapidly progressing inflammation. In contrast to early-onset models, in late-onset xlpra1 retinas a subset of the pro-inflammatory genes was highly upregulated long before any disease-related structural changes occurred, but was counterbalanced by an adequate anti-inflammatory response. Results point out to upregulated immune response accompanying disease progression in animal models of retinal degeneration, and to potential benefits of early anti-inflammatory therapy.

## Introduction

Retinitis pigmentosa (RP) is a heterogeneous group of inherited retinal degenerative diseases leading to photoreceptor cell death and severe vision loss. In RP, the initial defect occurs in the photoreceptors, either rods exclusively or rods and cones, followed by abnormalities in the adjacent retinal pigment epithelium (RPE) and deterioration of cones. Although RP is caused by mutations in over 60 different genes (http://www.retnet.org (latest entry 2017)), there now is increasing evidence supporting a prominent role for inflammation underlying disease pathogenesis [1–4].

When cells die *in vivo* they trigger an inflammatory response that can cause tissue damage, thereby contributing to the disease progression, and the manner of cell death and the resulting inflammatory responses are tightly linked processes. Necrotic cell death readily elicits a host inflammatory response while apoptotic cell death usually doesn’t provoke inflammation [5,6]. Moreover, apoptotic cells can stimulate macrophages to generate mediators such as interleukin 10 (IL10) or transforming growth factor beta (TGFB) that inhibit inflammation [5]. However, the statement that apoptosis is non-inflammatory is not always correct. Apparently, if the apoptotic cells are not rapidly cleared by phagocytes, over time they undergo a process known as secondary necrosis in which their membrane becomes permeable to macromolecules and incites an inflammatory response [5].

Under these conditions, release of damage-associated molecular patterns (DAMPs), including dsRNA, crystalline urea, extracellular ATP, cholesterol and degraded extracellular matrix components [7–11] can lead to activation of NLRP3 inflammasome, a key driver of inflammation that is formed after the oligomerization of NOD-like receptor NLRP3 and subsequent recruitment of the adaptor PYCARD and pro-caspase-1 [12,13]. Inflammasome activation leads to activation of caspase-1 (CASP1) and a consequent increase in caspase-1-dependent processing and secretion of the mature pro-inflammatory cytokines interleukin 1 beta (IL1B) and interleukin 18 (IL18). In addition, NLRP3 inflammasome activation leads to the induction of pyroptosis, a pro-inflammatory cell death pathway that eliminates the diseased cell [12,13]. In retinal disorders, retinal pigment epithelium (RPE), microglia and infiltrating macrophages are reported to be the cellular source of active caspase-1 [14–16], suggesting inflammasome-dependent inflammatory responses may contribute to disease progression.

**Fig 1:**
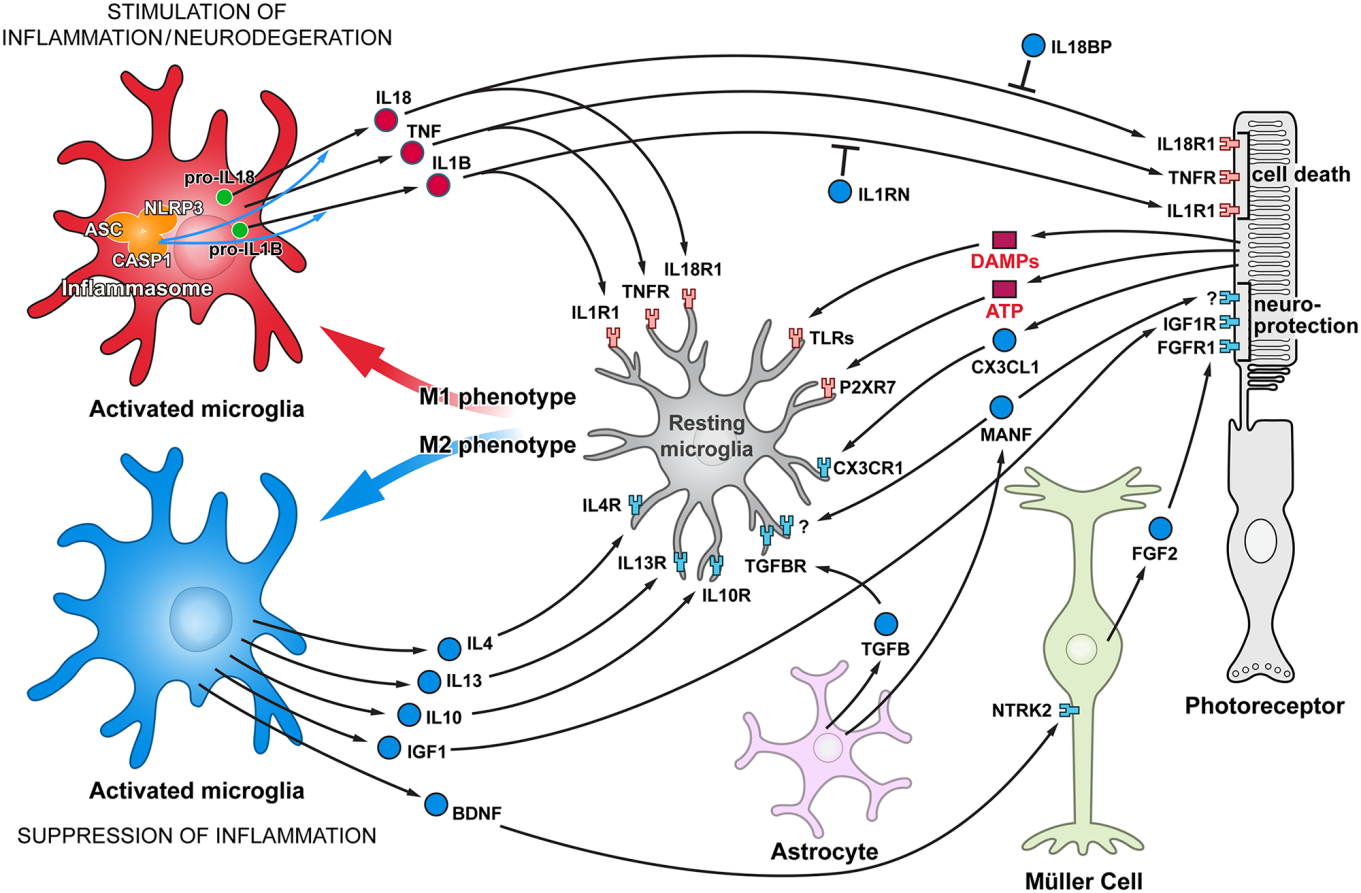
Functional cell-cell interactions in retina responsible for stimulating and suppressing inflammation. Schematic representation of glia-photoreceptor interactions in the retina. The connections are based on reported cellular interactions (for description, please see text). Microglia, the resident immune cells, are responsible for initiating an inflammatory response. Microglia activation in the degenerating retina is triggered by different DAMPs. Although some trigger molecules which activate microglia are predicted to be released from injured cells, e.g. ATP, other molecules have not yet been identified. Activated microglia are capable of acquiring diverse phenotypes that display different cell-surface and intracellular markers, secrete different factors, and exhibit different functions. Two extreme microglial phenotypes are shown: the classically activated (M1) phenotype that promotes a pro-inflammatory response, and the alternatively activated (M2) phenotype that facilitates an anti-inflammatory response. Microglia can control their own polarization through autocrine and paracrine mechanisms. M1-polarized microglial phenotype is promoted by several cytokines, including TNF, IL1B and IL18. On the other hand, microglia can be driven to M2 phenotype by stimuli like IL4, IL13, IL-10, TGFB, CX3CL1 and MANF. Microglia, using receptors and signals, are in constant communication with neurons and other retinal cells. In pathological conditions this tight communication between cells mediate adaptive responses within the retina. Comments: Three basic types of glial cell in the retina are shown: Müller cells, astroglia, and microglia phenotypes are resting or activated, M1 or M2. Pro-inflammatory cytokines/receptors are in red and anti-inflammatory/neuroprotective factors are marked in blue. In addition, since MANF receptor is not identified yet, it’s marked by “?”.

Microglia are resident monocytes in the retina and central nervous system that are functionally similar to macrophages, and possess all inflammasome derived machinery [17–19]. Microglia activation in the degenerating retina is thought to be triggered by the endogenous DAMPs, and activated microglia have two distinct phenotypes, classically activated (M1) and alternatively activated (M2) [20–22]. The M1 phenotype is characterized by the production of high levels of oxidative metabolites and pro-inflammatory cytokines, e.g. IL1B, IL18, IL6 and tumor necrosis factor (TNF). Alternatively, M2 cells secrete anti-inflammatory factors, neurotrophic molecules as well as low levels of pro-inflammatory cytokines; thus M2 cells are implicated in inhibiting inflammation and restoring homeostasis. During disease progression both M1 and M2, as well as their intermediate phenotypes, may be present. Several cytokines, including interleukins IL4 [23], IL13 [23], IL10 [24] and TGFB [25,26], promote the M2 phenotype. In addition, recent finding showed that microglial phagocytosis and activation underlying photoreceptor degeneration are managed by astrocyte derived neurotrophic factor (MANF) and fractalkine (CX3CL1) signaling [27,28]. As microglia are in constant communication with neurons and other retinal cells [28–30], the overall inflammatory response is governed by the balance between pro- and anti-inflammatory cytokines likely regulating disease severity and progression rate (Fig 1). Therefore, detailed knowledge of how inflammatory responses are regulated in the retina is critical for understanding the pathogenesis of complex diseases such as retinal degeneration (RD), and may provide an insight into prevention strategies and potential treatment options.

Here, we investigated inflammatory events in the retina in four canine models of RP. These included three non-allelic diseases characterized by abnormal photoreceptor development and early degeneration, i.e. early-onset disorders: rod cone dysplasia 1 (rcd1), early retinal degeneration (erd) and X-linked progressive retinal atrophy 2 (xlpra2) caused respectively by mutations in rod cyclic GMP phosphodiesterase β subunit (*PDE6B*) ([31,32]), Serine/Threonine Kinase 38 Like (*STK38L*) ([33,34]) and a 2-bp microdeletion in *RPGR* gene ([35]), and a post-developmental (late-onset) disorder, X-linked progressive retinal atrophy 1 (xlpra1), caused by a 5-bp microdeletion in *RPGR* [35]. The phenotypes associated with rcd1, xlpra2 and erd are quite severe and manifest themselves in the course of retinal development. In contrast, the disease phenotype of late-onset xlpra1 is manifested only after normal photoreceptor morphogenesis is completed, and disease progression is gradual [35,36].

We previously demonstrated that the initial phases of photoreceptor degeneration in the three early-onset disease models were accompanied by upregulation of several genes of the TNF superfamily and apoptotic pathways [37]. Although the study highlighted a potential role for signaling through the extrinsic apoptotic pathways in early photoreceptor cell death events, the study also showed upregulated gene expression of the two critical kinases that mediate TNF-dependent necroptosis (a programmed form of necrosis): RIP1/RIPK1 (receptor-interacting protein kinase 1) and RIP3/RIPK3 (receptor-interacting protein kinase 3) in rcd1 and xlpra2 during period of chronic photoreceptors death. These data suggest a possible contribution of an alternative cell death mechanism such as necroptosis [6] at later time points. Putative involvement of different types of cell death in pathogenesis of retinal degeneration in rcd1 and xlpra2 is also supported by sustained downregulation of X-linked inhibitor of apoptosis (XIAP) found in rcd1 throughout the peak of photoreceptors death and after, while in xlpra2 decreased XIAP levels were detectable only after the peak of cell death [37]. While XIAP has been thought to act primarily as a suppressor of cell-death proteases, recent study indicates that XIAP controls activation of RIPK3 that drives cell death and triggers inflammasome assembly and activation [38] as well as loss of XIAP can facilitate switch to TNF-induced necroptosis [39].

As the host immune response was not thoroughly investigated in the previous study [37], we could not determine if non-apoptotic cell death mechanisms also contributed to degeneration. Additionally no information is available on a mechanism underlying retinal degeneration in late-onset xlpra1 disease. To this end, we now have analyzed the complex pattern of the inflammatory response in these models, and expanded the analysis of immune response genes, including those encoding the inflammasome constituents, major components of the IL1B, IL18 and toll like receptor 4 (TLR4) pathways, several pro-inflammatory cytokines, and a subset of genes encoding neuroprotective and anti-inflammatory proteins. As involvement of anomalous epigenetic modifications in inflammatory diseases as well as in retinal neuron survival has been recently recognized [40–42], we also have characterized expression patterns of 13 genes encoding histone acetyltransferases (HATs) and histone deacetylases (HDACs). The complete list of 57 genes analyzed in this study with the corresponding descriptions is reported in S1 Table. The expression profiles were tested at different time points relevant to the disease progression.

## Materials and methods

### Ethics statement

The research was conducted in full compliance and strict accordance with the Association for Research in Vision and Ophthalmology (ARVO) Resolution on the Use of Animals in Ophthalmic and Vision Research. All the studies have been approved by the University of Pennsylvania Institutional Animal Care and Use Committee (IACUC).

### Tissue samples

Retinal samples used were those used in our previous studies [37,43,44]. Four different canine RD models were examined: three early-onset (rcd1, xlpra2 and erd) and one late-onset post-developmental photoreceptor degeneration (xlpra1).

As previously reported for rcd1, xlpra2, and erd, abnormalities and retinal degeneration begin early at different time points during retinal development [45–47]. Briefly, abnormal development of photoreceptors was recognizable as early as ~ 4 weeks (wks) of age for rcd1 and xlpra2 with the peak of photoreceptors death occurring at ~ 5 wks for rcd1 and ~7 wks for xlpra2 [44,45]. The fact that rcd1 is more severe is supported by the results of the TUNEL (terminal deoxynucleotidyl transferase (TdT) dUTP nick-end labeling) and ONL (outer nuclear layer) thickness analyses [37]. In erd, the mutation impairs the late phase of photoreceptor development. Examined erd mutant retinas showed maximum of TUNEL-positive photoreceptor cells approximately at 10.5–12 wks of age, however the ONL thickness is preserved until at least 14.1 wks [33,37] as there is compensatory proliferation of a subset of photoreceptor cells [37,48].

Similar to our previous study [37], rcd1, and xlpra2 retinas were examined in this study by gene and/or protein expression analysis at the most relevant disease-related phases of photoreceptors cell death: before cell death peak (*induction*; 3 wks); at cell death peak (*execution*; 5 and 7 wks) and during sustained but reduced cell death rate (*chronic cell death*; > 14 wks). In comparison to rcd1 and xlpra2, a more limited age sampling was available for erd: 9.6–12 wks (*execution* phase; gene expression analysis), 8–12 wks (western blot analysis), and 8–14.1 wks (immunohistochemistry).

In contrast to the early-onset disease models, the retina develops and functions normally in xlpra1, and photoreceptor degeneration begins after 11–18 months [36] showing remarkable phenotypic variability [36,43]. For the present study, three severity grades were defined: *Mild*-degeneration present only in the periphery after 1.5 years of age or later; *Moderate*-degeneration develops between 11 and 15 months of age; and *Severe*-photoreceptor degeneration presents centrally and peripherally earlier that 11 months of age [36,43]. For the present studies, xlpra1 retinas with established disease severity phenotypes (3–4 years old) were used for immunohistochemistry, and pre-degenerate, structurally normal (16 wks old) mutant retinas were used for gene expression and western blot analysis.

### RNA extraction and cDNA synthesis

Total RNA was isolated from canine tissues using a modified TRIzol and single chloroform extraction protocol as previously described [43]. First strand cDNA was synthesized in 20 μL reactions using the High Capacity RNA-to cDNA kit (Applied BioSystems, Foster City, CA) following the manufacturer’s recommendations.

### Relative quantification (ddCt) assay

All qRT-PCR (quantitative real-time polymerase chain reaction) experiments complied with the MIQE (Minimum Information for Publication of Quantitative Real-Time PCR Experiments [49]) guidelines. Gene expression was determined in age-matched 3, 5, 7, and 16 wks old normal, rcd1, and xlpra2-mutants, and at 9.6, 9.9 and 12 wks of age in erd mutants; each time point included three dogs/disease group. Additionally, six xlpra1 retinas at 16 wks of age were analyzed. Fig 1 illustrates the major genes putatively involved in retinal inflammation and neurodegeneration, or genes that act to suppress the inflammatory response. Specifically, 57 genes were analyzed by qRT-PCR, and the primer sequences are listed in S1 Table. In addition to the common set of 57 genes, four more genes (*TNF*, *IL6*, *IL10* and *FGF2*) were analyzed for xlpra1, as these were included in a previous study of the three early-onset disease models [37].

qRT-PCR was performed in a total volume of 25 μL in 96-well microwell plates on the Applied Biosystems 7500 Real-Time PCR System. All PCRs were performed using cDNA generated from 20 ng DNAase-treated RNA. The SYBR green platform was used for gene expression analysis using a primer concentration of 0.2 μM. The *TBP* gene expression level was used to normalize the cDNA templates and calculate of the ratio of diseased vs. normal using the ddCT method [50]. Amplification data were analyzed with the 7500 Software version 2.0.1 (Applied Biosystems). Unpaired t-test was used for statistical analysis. Genes with p<0.05 and fold changes (FC) > +/- 2 were considered differentially expressed.

### Fluorescent immunohistochemistry (IHC)

The procedures used for tissue preparation, and sectioning were previously described [45]. Cryosections were washed and treated with the primary antibodies in PBS solution, 3% normal horse serum, 1% BSA and 0.3% Triton X-100 overnight followed by incubation with appropriate fluorescent secondary antibodies (Alexa Fluor Dyes, 1:300; Molecular Probes, Eugene, OR). Validated primary antibodies used for IHC are listed in S2 Table. Primary antibodies that were tested but failed to detect the specific antigen by IHC or western blot are listed in S3 Table. Staining was examined by epifluorescence microscopy with a Zeiss Axioplan microscope (Carl Zeiss Meditech, Oberkochen, Germany). Images were digitally captured (Spot 4.0 camera; Diagnostic Instruments, Inc., Sterling Heights, MI), and imported into a graphics program (Photoshop; Adobe, Mountain View, CA) for display. IHC was done on retinal section in 3, 5, 7 and 16 wks rcd1 and xlpra2; 20–23 wks and 3–4 years old xlpra1; 8 and 14.1 wks erd. Note that although the entire retinal expanse was examined in the immunolabeled sections, the images used in the illustrations were taken at 6,000 μm central to the ora serrata; this area is approximately the midpoint between the edge of the optic disc and the ora serrata.

### Western blot analysis

Western blots were carried out essentially as previously described [43]. The primary antibodies used in for western blot are listed in S2 Table. Protein concentrations were determined by BCA Protein Assay (Thermo Fisher Scientific, Rockford, IL), and equal micrograms of protein analyzed. Quantification of proteins on western blot was carried out with Li-COR Odyssey Fc software. Briefly, experiment was done a minimum of three times for each disease (rcd1, erd, xlpra2, and xlpra1) and time points analyzed. After scanning as done by Li-COR Odyssey Fc, the resulting relative fluorescence value from each band was normalized with respect to the housekeeping protein actin beta (ACTB). Next, normalized values from each disease in every experiment were represented as fold-changes as compared to the normal tissue value. For each disease, the mean value and standard error were calculated and graphed. Student t test (95% confidence intervals) was used for statistical analysis. Western blot was carried out in total retinal protein extracts in 7 and 16 wks old rcd1 and xlpra2; 8 and 12 wks erd; 16 wks xlpra1 as well as age-matched normal control.

## Results

### Modulation of inflammatory gene expression in RD

#### Early-onset diseases

We have used the schematic diagram (Fig 1) to display some of the major genes and pathways putatively involved in retinal inflammation and neurodegeneration, or genes that act to suppress the inflammatory response. To characterize inflammatory events in the retina, we have analyzed 23 pro-inflammatory immune response genes (see S1 Table (group 1). Those genes that were either differentially expressed or unaffected by the disease process were further filtered and shown in Table 1 and S4 Table, respectively.

**Table 1:** Comparative analysis of differentially expressed (DE) genes by qRT-PCR in study models: Pro-inflammatory immune response group. DE genes (p<0.05 and FC≥+/-2) between rcd1, xlpra2, erd and xlpra1 mutants compared to normal at different ages.

DE genes	FC rcd1 vs. normal	FC xlpra2 vs. normal	FC erd vs. normal	FC xlpra1 vs. normal
	***3 wks***	***3 wks***		
*IL1R1*	2.0	n.s.*		
*IL18*	2.2	n.s.		
*CXCL8*	-2.7	n.s.		
	***5 wks***	***5 wks***		
*IL1R1*	3.1	n.s.		
*IL1R2*	2.4	n.s.		
*IL18*	2.2	n.s.		
*CXCL8*	-2.0	n.s.		
	***7 wks***	***7 wks***		
*NLRP3*	3.9	n.s.		
*CASP1*	2.8	n.s.		
*PYCARD*	6.7.	2.1		
*IL1B*	8.6	n.s.		
*IL1R1*	9.0	2.7		
*IL1R2*	2.7	n.s.		
*IL18*	7.4	2.0		
*TLR4*	5.2	n.s.		
*IRAK4*	2.6	n.s.		
*P2RX7*	2.1	n.s.		
*CXCL8*	2.0	n.s.		
*CSF1R*	2.4	n.s.		
*CD74*	3.2	n.s.		
	***16 wks***	***16 wks***	***9*.*6–12 wks***	***16 wks***
*NLRP3*	8.3	4.7	2.0	n.s.
*CASP1*	14.4	13.7	2.6	n.s.
*PYCARD*	10.3	3.9	2.6	n.s.
*IL1B*	5.6	n.s.	n.s.	55.7
*IL1R1*	10.3	3.5	4.2	n.s.
*IL1R2*	8.4	n.s.	n.s.	n.s.
*IL18*	12.8	5.6	2.5	n.s.
*IL18R1*	11.4	10.1	2.6	n.s.
*TLR4*	5.4	2.7	2.0	15.2
*MYD88*	3.3	2.0	n.s.	n.s.
*IRAK4*	5.2	6.3	n.s.	n.s.
*P2RX7*	2.4	n.s.	n.s.	2.5
*CXCL8*	3.7	n.s.	-3.6	n.s.
*SYK*	n.s.	2.7	n.s.	n.s.
*CSF1R*	4.4	3.9	n.s.	2.4
*CD200R*	4.2	4.2	n.s.	n.s.
*CD74*	10.8	4.9	n.s.	n.s.

Notes:

*n.s. = not statistically significant

At the time points tested, the highest number of differentially expressed genes were found in rcd1, in agreement with the observation that this disease is more aggressive and earlier in onset than the others. Of the 23 genes tested, eight encoded inflammasome components (*NLRP3*, *CASP1* and *PYCARD*) as well as inflammasome substrates (*IL1B* and *IL18*) along with their receptors (*IL1R1*, *IL1R2* and *IL18R1*). The earliest changes in gene expression were noted in rcd1 at 3 wks (*induction* phase of the disease), and were characterized by slight upregulation of *IL1R1* and *IL18*. The number of differentially expressed genes in rcd1 gradually increased from 3 wks towards 5 wks and 7 wks (*execution* phase), and the entire subset of eight genes studied was highly upregulated by 16 wks (*chronic cell death* phase).

Although the pattern of differential gene expression in xlpra2 at 16 wks (*chronic cell death* phase) was similar to rcd1 at the same age, first changes at mRNA level were present at 7 wks, and even then, not all genes upregulated in rcd1 were comparably altered in xlpra2. More specifically, mRNA levels of *IL1R1*, *IL18* and *PYCARD* were slightly elevated at 7 wks, and at 16 wks these three genes as well as *NLRP3*, *CASP1* and *IL18R1* were significantly upregulated. The same six genes were upregulated in erd retinas between 9.6 wks and 12 wks of age (*execution* phase), with a maximal 4.2-fold increase for *IL1R1*, while *IL1B* and *IL1R2* were not differentially expressed.

Next, we examined the expression of a group of genes involved in inflammasome activation (*P2RX7*, *SYK*, *TLR4*) [5], and common components of IL1B, IL18 and TLR4 pathways (*MYD88*, *IRAK4* and *TRAF6*). *TRAF6* was not differentially expressed in any early-onset disease models. However, in rcd1, we found *TLR4*, *IRAK4* and *P2RX7* were increasingly upregulated at 7 wks (*execution*) and 16 wks (*chronic cell death*), with an overall 5.4-fold increase in *TLR4* at 16 wks. Also *MYD88* was upregulated in rcd1 (3.3-fold) but only at 16 wks. In xlpra2, four genes (*SYK*, *TLR4*, *MYD88* and *IRAK4*) were upregulated only at 16 wks (*chronic cell death*), with a maximum 6.3-fold increase in *IRAK4*. Of six genes analyzed in erd (9.6–12 wks, *execution* phase), only *TLR4* gene expression was upregulated by 2.0-fold.

Lastly, we evaluated expression level of the remaining subset of nine genes that mediate pro-inflammatory immune response, including *CXCL8*, *TXNIP*, *PTGES*, *CD74*, *CSF1R*, *CD200R*, *VEGFA*, *FLT1* and *KDR*. *TXNIP*, *PTGES*, *VEGFA* and its two receptors (*FLT1* and *KDR*) were not differentially expressed in any of the three models, suggesting low functional role of the VEGF (angiogenesis pathway) or prostaglandin E (acute inflammatory response) pathways in pathogenesis of retinal degeneration. The remaining three genes that are involved in microglia/macrophages activation and survival, *CXCL8*, *CD74* and *CSF1R*, were only slightly upregulated in rcd1 at 7 wks (*execution*), but, along with *CD200R*, were prominently upregulated at 16 wks (*chronic cell death*) with an overall maximal 10.8-fold increase for *CD74*. Notably, in rcd1 the chemokine gene *CXCL8* was downregulated at 3 and 5 wks. On the other hand, expression of these three genes in xlpra2 was elevated only at 16 wks, while the rest of the genes examined were unchanged. Lastly, in erd, just one out of nine analyzed genes (*CXCL8*) was differentially expressed, but in this case the expression was decreased by 3.6-fold.

#### Gene expression changes in late-onset disease

The same set of 23 genes was analyzed in the late-onset xlpra1 (Table 1 and S4 Table). In pre-degenerate mutant retinas at 16 weeks of age, a time when the retina is structurally normal [36] but rod opsin is mislocalized [44], we observed strong upregulation of *IL1B* and *TLR4* along with more subtle expression increases for *P2RX7* and *CSF1R*. There were no differences in expression of the remaining 19 genes. Because of the magnitude of *IL1B* and *TLR4* upregulation, we increased the sample size from three to six to verify our results. This analysis confirmed the initial findings and showed that *IL1B* and *TLR4* were upregulated by 55.7-fold and 15.2- fold, respectively. Finally, since the mRNA levels of the pro-inflammatory cytokines *TNF* and *IL6* has been previously reported to be elevated in early-onset rcd1, xlpra2 and erd [37], we also examined mRNA levels of *TNF* and *IL6* in late-onset xlpra1, and found them unchanged.

Together, these findings suggest prompt initiation and sustained increase in host immune response via activation of broad range of pro-inflammatory genes expression along the timeline of photoreceptor death in all early onset RD models. In terms of magnitude of differential expression of pro-inflammatory genes at the peak of photoreceptors death erd occupies intermediate position between rcd1 and xlpra2. In contrast, in late-onset xlpra1, strong upregulation of several pro-inflammatory genes long precedes the onset of photoreceptor degeneration.

### Inflammatory protein expression in RD models

#### Early- and late-onset diseases

**Fig 2:**
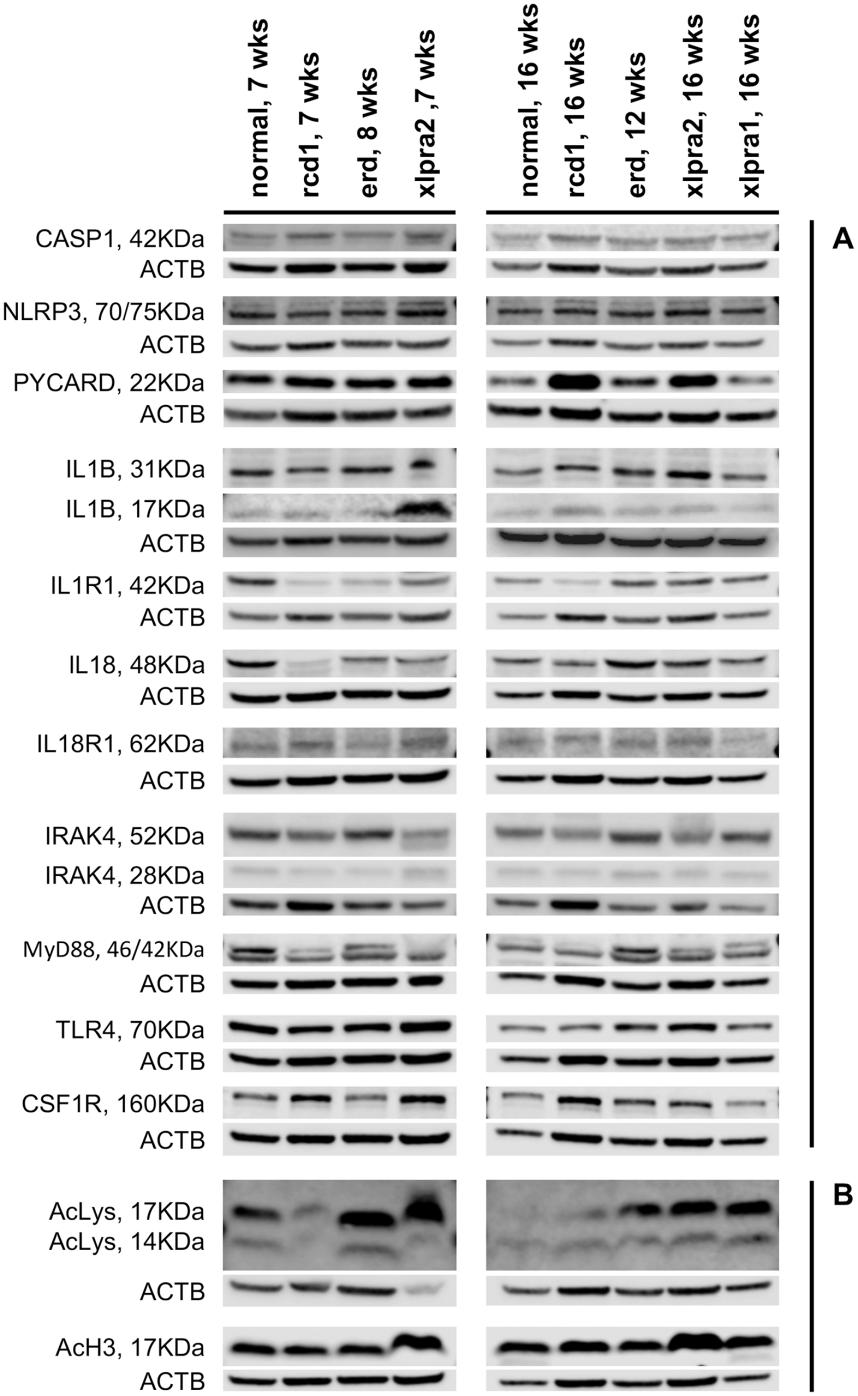
Western blot analysis of pro-inflammatory (A) and histone acetylation proteins (B) in study models of retinal degeneration. (A) Representative western immunoblot was performed for the expression of 11 proteins involved in pro-inflammatory signaling in total retinal protein extracts for normal and mutant retinas at 7 wks (rcd1, xlpra2), 8 wks (erd), 12 wks (erd), and 16 wks (rcd1, xlpra2, xlpra1). The following proteins were analyzed: inflammasome components (CASP1, NLRP3 and PYCARD), inflammasome substrates (IL1B and IL18) and their receptors (IL1R1 and IL18R1), inflammasome receptor (TLR4), common components of IL1B-, IL18- and TLR4-pathways (MYD88 and IRAK4) and macrophages expressing protein (CSF1R). (B) Level of histone acetylation in retinal protein extracts from the same four disease models was evaluated with acetylated-Lysine and acetyl-Histone H3 antibodies at the indicated time points.

**Fig 3:**
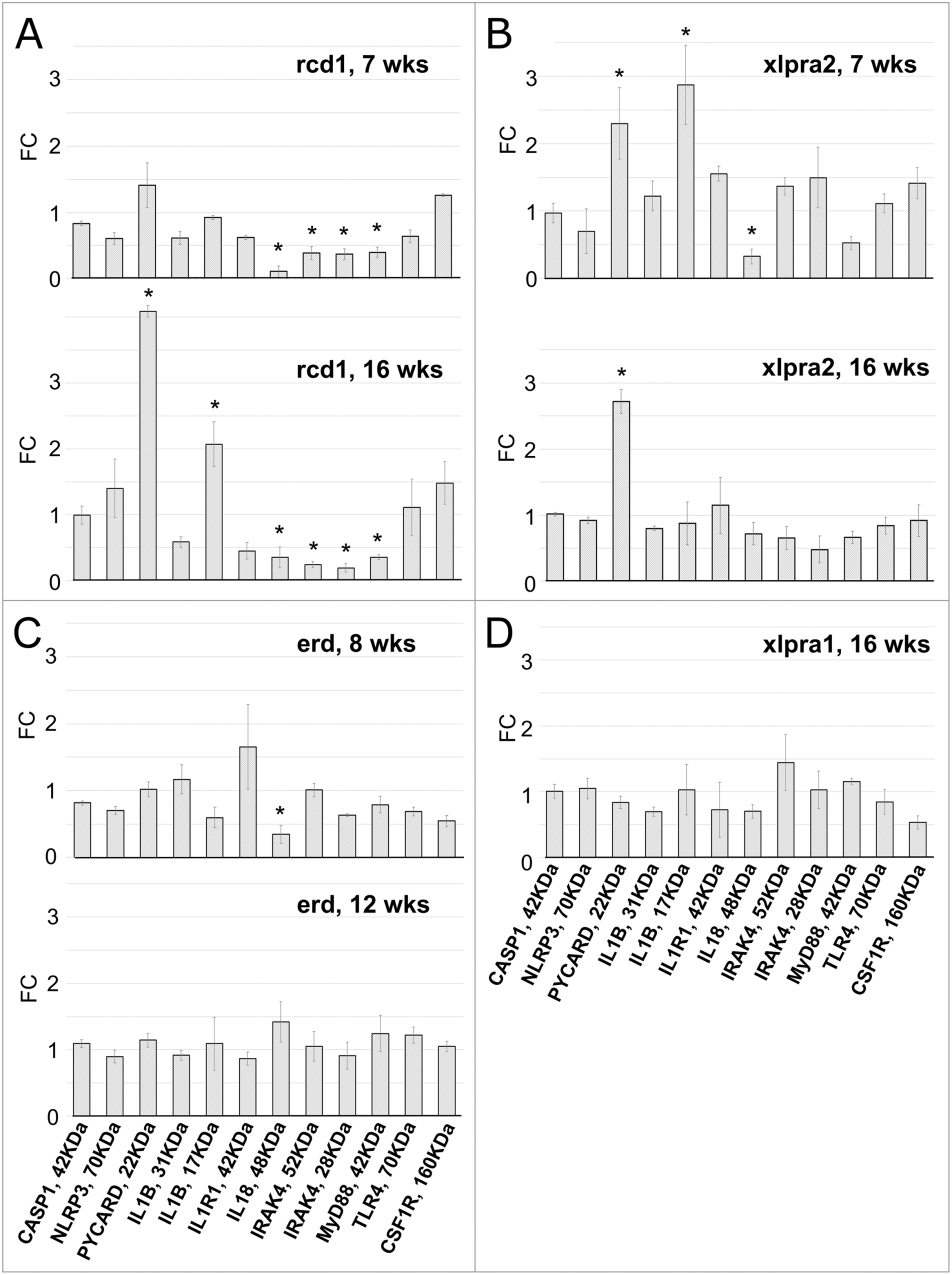
Protein quantification in retinas of rcd1 (A), xlpra2 (B), erd (C) and xlpra1 (D) models. Quantification of pro-inflammatory proteins on western blots (Fig 2A) normalized against the corresponding housekeeping protein (ACTB) was done using Li-COR Odyssey Fc and represented as fold-changes compared to the normal tissue values. Differences in relative fluorescence (Y-axis) for the proteins analyzed (X-axis) show CASP1, NLRP3, IL1B-precursor, IL1R1, TLR4 and CSF1R in disease (rcd1, xlpra2, erd, xlpra1) were similar to normal levels at all ages. In contrast, two proteins were increased over normal levels: mature ILIB-17 KDa (7 wks xlpra2, 16 wks rcd1) and PYCARD (7 wks xlpra2, 16 wks xlpra2, 16 wks rcd1). Moreover, three proteins were below normal levels: MYD88 (7 wks rcd1, 16 wks rcd1), IRAK4 (7 wks rcd1, 16 wks rcd1) and IL18-precursor (7 wks rcd1, 16 wks rcd1, 8 wks erd, 7 wks xlpra2). * indicates significance level 5% (p<0.05).

Western blot analysis: immune response gene expression is tightly regulated at transcriptional and translational levels, but there can be a poor correlation between mRNA expression and the abundance of its encoded protein [51,52]. It is thought that control of gene expression, mRNA stability and protein translation all act in concert to fine-tune and modulate the initiation, duration and magnitude of the innate immunity inflammatory response [52]. To examine whether the increased transcription of the inflammatory genes resulted in a concomitant increase in protein expression and activation of the inflammasome multiprotein complex in the studied disease models, we next determined the relative amounts of 11 proteins (CASP1, NLRP3, PYCARD, IL1B, IL1R1, IL18, IL18R1, MYD88, IRAK4, TLR4 and CSF1R) in rcd1 and in xlpra2 retinas at 7 and 16 wks, in erd retinas at 8 and 12 wks, and in xlpra1 retinas at 16 wks using western blot analysis (Figs 2 and 3).

Despite the observed increases in gene expression, the variations in protein expression were not statistically significant for CASP1, NLRP3, IL1B-precursor, IL1R1, TLR4 and CSF1R in any of the four disease models. However, several other proteins were differentially expressed in early-onset models, rcd1 and xlpra2. In rcd1 we observed a 4.1-fold increase in PYCARD protein levels accompanied by an increase in the mature form of IL1B (17 KDa) at 16 wks, indicative of inflammasome-dependent caspase-1 activation. The protein expression levels of MYD88, IRAK4 and IL18-precursor (48 KDa homodimer [53]) were drastically decreased in rcd1, while mature 18 KDa IL18 was not detected at all.

In xlpra2, we found increased PYCARD levels at 7 (2.3-fold) and 16 wks (2.7-fold), while the mature IL1B was upregulated only at 7 wks. The IL18-precursor was downregulated by 3.3-fold at 7 wks but returned to normal control level at 16 wks. The variations in MYD88, IRAK4 expression were not significant at either 7 wks or 16 wks, while mature IL18 was again undetectable, similar to what was found in rcd1. There was no apparent change in protein expression in erd at 12 wks, or in late-onset xlpra1 at 16 wks. However, the IL18-precursor was significantly decreased in erd at the earlier time point studied, similar to what was observed in rcd1 and xlpra2.

**Fig 4:**
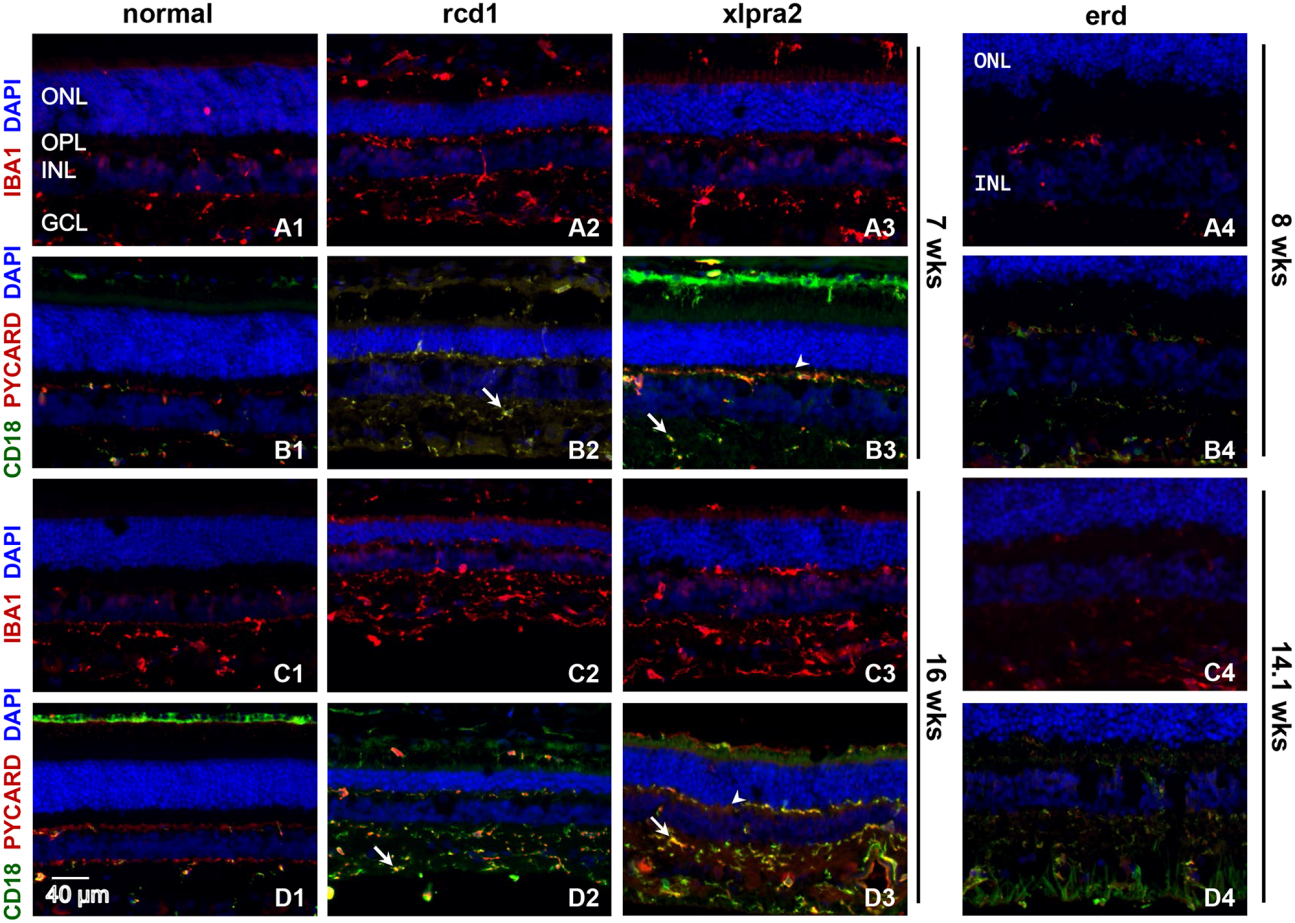
Retinal localization of selected pro-inflammatory proteins in early-onset disease models. Immunolabeling of normal and early-onset disease (rcd1, erd, xlpra2) retinas at various ages using IBA1, PYCARD and CD18 antibodies. IBA1 (red) labeling, a well-known marker of reactive microglia, increases in rcd1 and xlpra2 at 7 and 16 wks (A2, A3, C2, C3) when compared to normal control (A1, C1), suggesting microglial activation. IBA^+^ cells demonstrate migration of microglia from inner retina towards the outer retinal layers of rcd1 (A2, C2) and xlpra2 (A3, C3) at all ages tested. Additionally, double immunolabeling of the same retinal samples were also done with microglia/macrophage marker CD18 (green) and the inflammasome component PYCARD (red) antibodies. PYCARD is expressed primarily in CD18^+^ positive cells, as shown by labeling overlapping (B2-B4, D2-D4) thus demonstrating inflammasome component expression specifically in retinal microglia, albeit in two distinct subpopulations; CD18^+^/PYCARD^+^ (yellow; arrow) and CD18^-^/PYCARD^+^ (red; arrowhead) cells. PYCARD expression is observed at all studied ages in diseased (B2-B4, D2-D4) and normal (B1, D1) retinas, and is significantly upregulated in rcd1 and xlpra2, especially at 16 wks of age. Conversely, IBA1 staining in erd retinas at 8 wks (A4) and 14.1 wks (C4) do not significantly change when compared to normal (A1, C1) indicating no additional microglial activation is occurring. Similarly, PYCARD/CD18 staining in erd at 8 and 14.1 wks remains the same as normal retinas. Although two subpopulations of CD18/PYCARD cells are also observed in erd retinas, increase in microglia migration to the outer retina layers as seen in rcd1 and xlpra2 was noticeably absent. ONL = outer nuclear layer; OPL = outer plexiform layer; INL = inner nuclear layer; GCL = ganglion cells layer. Scale bar 40 μm.

Immunohistochemical characterization: we first characterized the severity of retinal inflammation in the early onset RD models by analyzing the expression pattern of several proteins involved in the inflammatory response in normal and mutant retinas. First, we examined expression of the allograft inflammatory factor-1, also known as IBA1, a microglia/macrophage marker that is upregulated during the activation of these cells [54]. Analysis of IBA1 labeling in rcd1 and xlpra2 showed significant upregulation of this microglia marker from 7 wks onwards (Fig 4A1–4A3 and 4C1–4C3) but not in erd at 8 and 14.1 wks (Fig 4A4 and 4C4). Inflammasome component PYCARD immunolabeling showed significant overlap with MHC class II expression by beta2 integrin (CD18) (immune surveillance marker) expression patterns, but the protein also was present in CD18 negative microglia, mostly in the inner retina. PYCARD immunolabeling was visibly increased in rcd1 and xlpra2 at 16 wks compared to 7 wks (Fig 4D1–4D3). The intensity of PYCARD labeling in erd at 8 and 14.1 wks was not changed and remained close to normal (Fig 4B4 and 4D4). In addition, we have observed microglia/macrophage migration toward the outer retinal layers and increased numbers of IBA^+^/CD18^+^ cells as early as at 3 wks in rcd1 and at 5 wks in xlpra2 (S1 Fig). In contrast to rcd1 and xlpra2, where active migration of retinal microglia was first noticeable prior to the peak of photoreceptors cell death and continued thereafter at all studied ages in disease retina, in erd no increase in microglia migration was detected neither at 8 wks nor at 14.1 wks of age. These findings clearly demonstrate that the immune response is initiated early during the induction phases of retinal degeneration in both rcd1 and xlpra2 models. As expected, migration and activation of retinal microglia positively correlate with thinning of ONL in these two diseases. Conversely, intraretinal migration and activation of microglia were noticeably absent in erd at 14.1 wks, a time when the thickness of the ONL is still normal [33].

**Fig 5:**
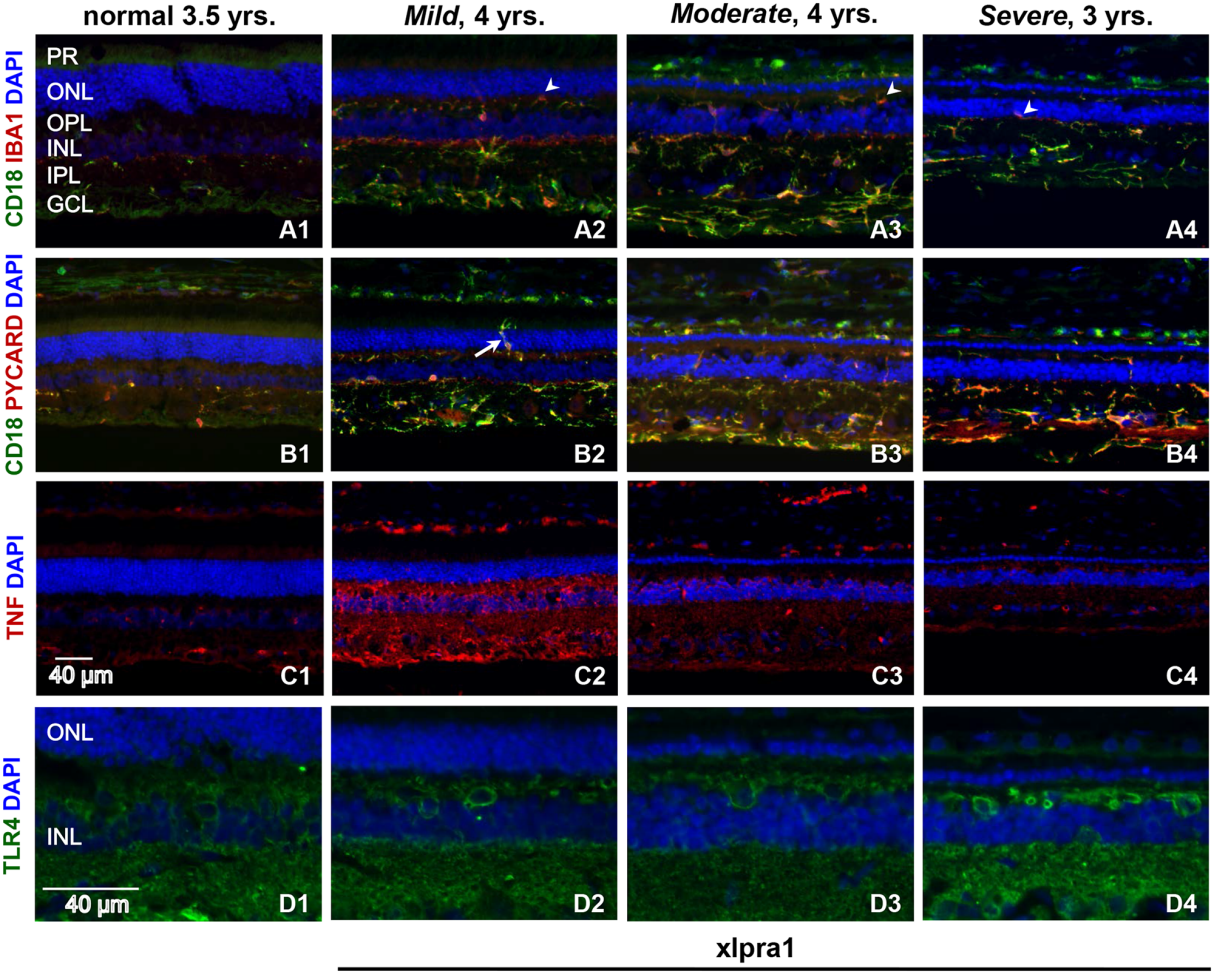
Retinal localization of pro-inflammatory proteins in late-onset xlpra1-affected retinas with different disease severity phenotypes. Immunolabeling of normal and late-onset disease (xlpra1) retinas with *Mild*, *Moderate* and *Severe* phenotypes was done using IBA1, CD18, PYCARD, TNF and TLR4 antibodies. Double immunolabeling with microglial markers CD18 (green) and IBA1 (red) antibodies was done (A1–A4) to describe the phagocytes response characterized by their morphological changes and intraretinal migration. IBA1 and CD18 show significant co-localization in xlpra1 retinas displaying a well-developed disease phenotype that includes reduction of ONL (A2-A4). Both IBA1 and CD18 immunolabeling are robustly upregulated in disease retinas although highest expression is seen in *Moderate* severity. Double staining (A1-A4) of IBA1 and CD18 shows three subpopulation of phagocytes are seen in xlpra1 retina: CD18^+^/IBA1^+^ (yellow), CD18^+^/IBA1^-^ (green) and CD18^-^/IBA1^+^ (red). Additionally, aggressive invasion of retinal layers by activated microglia/macrophages (arrowhead) is noticeable (A2-A4), particularly at *Mild* stage. Double immunolabeling of CD18 (green) with PYCARD (red) antibodies (B1-B4) shows PYCARD expression in CD18^+^ positive cells (yellow), with upregulation of both proteins in affected retinas. Robust infiltration of ONL by activated microglia/macrophages (B2) is very prominent in *Mild* stage (arrow). TNF labeling in normal and diseased retinas (C1-C4) shows TNF present in all retinal layers following the same pattern at all disease stages analyzed, however in *Mild* disease labeling signal was highest while intensity decreases as disease progresses. TLR4 labeling is present in all layers of normal and diseased retinas with labeling higher in *Severe* phenotype. PR = photoreceptors; ONL = outer nuclear layer; OPL = outer plexiform layer; INL = inner nuclear layer; IPL = inner plexiform layer; GCL = ganglion cells layer. Scale bar 40 μm.

We next characterized the retinal response to disease by comparing expression pattern of microglial markers IBA1 and CD18, PYCARD, TNF (a well-known master regulator of apoptosis and necroptosis) and TLR4 (a sensor for a danger signal that alert the immune system to tissue) in the late-onset xlpra1 disease model (Fig 5B1–5B4). As noted previously, photoreceptor degeneration begins after 11–18 months of age, and shows remarkable variability in disease progression although the factor(s) determining this variability are unknown [43,55]. To investigate the pathogenic process that accompanies xlpra1, we used retinas with established disease, and examined the expression patterns of these proteins as well as their colocalization to various cell types in retinas with different degrees of disease severity [36,43].

Notably, IBA1 labeling is increased in xlpra1, and this increase is associated with phenotype severity (Fig 5A1–5A4). In agreement with previously reported observations of substantial heterogeneity in retinal population of resident microglia/macrophages characterized by their differences in morphology, antigen expression and distribution [56,57], double staining for IBA1 and CD18 showed three sub-populations of cells expressing IBA1, CD18 or both proteins (Fig 5A1–5A4). However, the proportion of IBA1^+^/CD18^+^ cells was increased in retinas with a more severe disease phenotype compared to normal. Notably, in xlpra1, immunolabeling of IBA1 revealed that photoreceptor degeneration induced a massive response in phagocytes that was characterized by distinct morphological changes and intraretinal migration.

Immunolabeling with PYCARD showed increased expression of this protein in mutant retinas compared to normal, and double labeling with PYCARD and CD18 identified PYCARD expression in microglia/macrophages cells at all disease phases (Fig 5B1–5B4). Similar to the previously published data in rcd1, xlpra2 and erd [37], TNF labeling was most intense in the *Mild* phenotype, and gradually decreased with disease severity characterized by severe photoreceptor loss (Fig 5C1–5C4). Such pattern of TNF labeling differs previous observations in rcd1 and xlpra2 [37] which showed increased TNF immunolabeling with disease progression.

As it’s seen on (Fig 5D1–5D4) TLR4 localization and staining intensity was similar in normal and xlpra1 retinas with *Mild* and *Moderate* disease phenotype, however, more intense distribution and expression of the TLR4 labeling was observed in the *Severe* phenotype. TLR4 was detected in inner retina, inner nuclear layer, outer plexiform layer, and at a lower level in RPE. Rod opsin mislocalization manifests long before any structural changes appear in xlpra1, and can be detected as early as 20 wks of age [44]. We took advantage of the patchy retinal degeneration that occurs in heterozygous carrier females to determine if diseased retinal patches, evident by areas of rod opsin mislocalization, were associated with signs of macrophage/monocyte infiltration. We observed redistribution of CD18^+^ cells from inner toward outer retinal layers in xlpra1 carriers (S2 Fig), and CD18^+^ cells concentrated in areas of rod opsin mislocalization, suggesting that this is an early retinal response to the disease (S2 Fig).

### Expression profiles of neuroprotective and anti-inflammatory genes

The overall effect of the immune response in diseased retina is determined by the balance of pro-inflammatory and anti-inflammatory/neuroprotective factors. To this end, we examined expression of a subset of selected genes involved in anti-inflammatory response and neuroprotection. The expression levels of 21 genes were evaluated in the four RD models, and the results are presented in Table 2 and S5 Table. The panel was composed of a subset of genes encoding neurotrophic factors and their receptors (*MANF*, *LIF*, *FGFR1*, *CNTFR*, *NTRK2*, *NTRK3* and *IGF1*), regulators of cells proliferation, differentiation and growth (*TGFB1*, *TGFB2*, *PDGFA*, *PDGFB*, *PDGFRA* and *PDGFRB*), anti-inflammatory factors (*IL4*, *IL13*, *CX3CL1*, *CX3CR1*, *EGF* and *EGFR*) and inhibitors of IL1B and IL18 signaling (*IL1RN* and *IL18BP*, respectively). It should be noted that while we placed *EGF* and *EGFR* into the anti-inflammatory group, EGF/EGFR-signaling may act as either anti-inflammatory or pro-inflammatory, depending upon the local tissue conditions [58,59].

**Table 2:** Comparative analysis of differentially expressed genes in study models: Neuroprotective and anti-inflammatory group. DE genes between rcd1, xlpra2, erd and xlpra1 mutants compared to normal at different ages.

DE genes	FC rcd1 vs. normal	FC xlpra2 vs. normal	FC erd vs. normal	FC xlpra1 vs. normal
	***5 wks***	***5 wks***		
*LIF*	6.1	n.s.		
	***7 wks***	***7 wks***		
*CX3CR1*	2.1	n.s.		
*LIF*	29.1	5.0		
*IL4*	2.4	n.s.		
*EGF*	2.6	n.s.		
*EGFR*	3.4	n.s.		
	***16 wks***	***16 wks***	***9*.*6–12 wks***	***16 wks***
*IL1RN*	20.6	10.8	2.0	n.s.
*CX3CL1*	2.9	n.s.	n.s.	n.s.
*CX3CR1*	11.4	4.2	n.s.	n.s.
*PDGFA*	2.4	n.s.	4.9	n.s.
*PDGFRB*	3.8	n.s.	2.0	-2.1
*LIF*	8.7	17.4	n.s.	3.0
*TGFB1*	3.7	4.5	2.8	n.s.
*IL4*	2.1	n.s.	n.s.	14.9
*EGF*	2.8	n.s.	n.s.	n.s.
*EGFR*	9.0	2.6	n.s.	n.s.
*FGFR1*	6.1	n.s.	n.s.	n.s.
*FGF2*				3.9

The expression of ten genes (*IL18BP*, *PDGFB*, *PDGFRA*, *CNTFR*, *MANF*, *TGFB2*, *IL13*, *NTRK2*, *NTRK3* and *IGF1*) remained similar to normal in all four diseases. The remaining eleven genes (Table 2) were upregulated in rcd1 at 16 wks, with an overall maximal 20.6-fold increase in expression of the IL1B antagonist gene, *IL1RN*. In rcd1, the expression of five genes (*CX3CL1*, *LIF*, *IL4*, *EGF*, and *EGFR*) was elevated at 7 wks, while *LIF* was also upregulated as early as at 5 wks. Similar to rcd1, *IL1RN*, *CX3CL1*, *LIF*, *TGFB1* and *EGFR* were upregulated in xlpra2 at 16 wks, while *LIF* was increased at 7 wks. In erd, *IL1RN*, *TGFB1* and two of PDGF pathway genes (*PDGFA* and *PDGFRB*) exhibited mild to moderate upregulation at 9.6–12 wks.

The same set of genes was analyzed at 16 wks late-onset xlpra1, a pre-degeneration time point. Of 21 genes examined, only three genes studied showed significant differences in expression. Of these three, *PDGFRB* was significantly downregulated by 2.1 folds, whereas *LIF* and *IL4* were elevated, with *IL4* being the highest 14.9-fold increase. Also, two additional genes, anti-inflammatory cytokine *IL10* and neuroprotective factor *FGF2*, were analyzed in xlpra1. These two genes were previously studied in rcd1, erd and xlpra2 [37], though never in xlpra1. In this study we found *IL10* expression remained close to normal, but *FGF2* expression was upregulated by 3.9-fold.

Altogether, our results point to an active dynamic interaction between pro- and anti-inflammatory/neuroprotective pathways in all RD models. However, there is a common tendency for a delayed anti-inflammatory/neuroprotective response following initiation of the pro-inflammatory gene expression in all early-onset RD models. In contrast, there appears to be a prompt initiation of anti-inflammatory/neuroprotective response in the early phases of the disease in late-onset xlpra1, long before there are any significant structural alterations or photoreceptor cell death. This results in a more balanced dynamic regulation between pro- inflammatory and anti-inflammatory/neuroprotective genes in late-onset xlpra1.

### Expression of Histone deacetylases and acetyltransferases in RD models

Several studies have related the response of activated immune cells to histone modifications of specific inflammatory genes [42,60–62]. In this study, we examined the expression profiles in the four disease models of a representative subset of HDACs and HATs by comparing mRNA transcripts levels of nine HDACs of class I (*HDAC1*, *HDAC2* and *HDAC3*), class II (*HDAC4*, *HDAC5*, *HDAC6* and *HDAC9*) and class III (*SIRT1* and *SIRT2*) as well as four HATs (*KAT21*, *EP300*, *CREBBP* and *TAF1*). Results are shown in the S6 Table. In addition, we evaluated the level of histone acetylation in all four retinal disease models by western blot analysis using antibodies against acetylated-Lysine and acetyl-Histone H3.

In early-onset diseases, there were no significant changes in mRNA expression levels; out of total nine HDAC genes, only *HDAC4* and *HDAC5* were mildly downregulated in xlpra2 at 7wks, and *HDAC9* was mildly downregulated in erd at 9.6–12 wks. Of the 13 genes analyzed, only *EP300* was downregulated by 2.0-fold in late-onset xlpra1.

**Fig 6:**
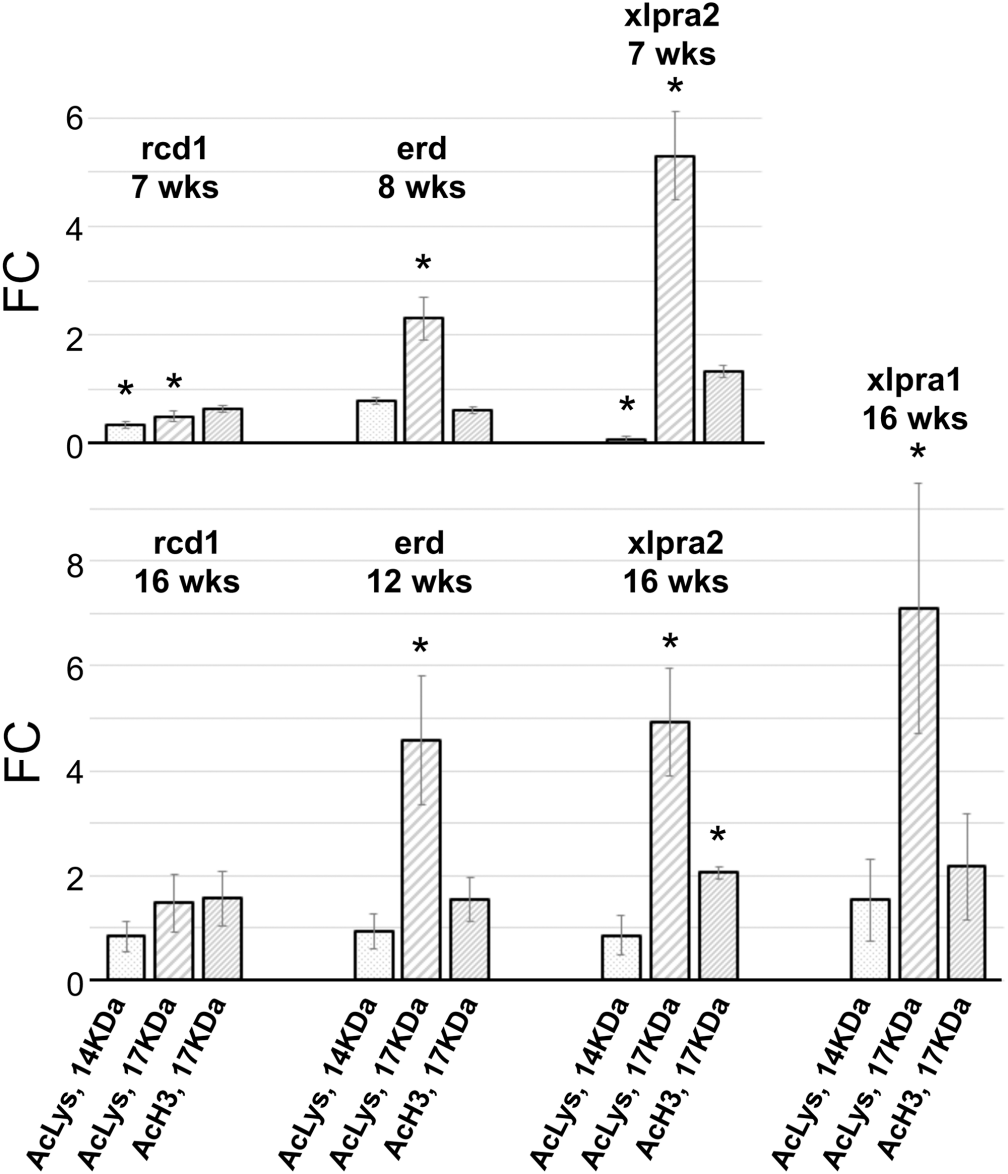
Quantification of histone acetylation level in rcd1, xlpra2, erd and xlpra1 retinas. Quantification of histone acetylation on western blots (Fig 2B) normalized against the corresponding housekeeping protein (ACTB) was done using Li-COR Odyssey Fc and represented as fold-changes compared to the normal tissue values. Differences in relative fluorescence (Y-axis) for the proteins analyzed (X-axis) show 14 KDa-Acetyl-Lysine levels decrease in rcd1 and xlpra2 at 7 wks but remain the same as normal in all other samples. 17 KDa-Acetyl-Lysine was increased in all samples except in 7 wks rcd1 (decreased) and 16 wks rcd1 (same as normal). In contrast, acetylated H3 histone shows mostly unchanged pattern, except in 16 wks xlpra2 where it was increased. * indicates significance level 5% (p<0.05).

Western analysis with acetylated-Lysine antibody detected ~17 KDa and ~14 KDa bands that corresponded to the reported molecular weight of different histones (Figs 2B and 6). Interestingly, there was a unique pattern of intensity of 17 KDa and 14 KDa bands that was specific to each disease. For instance, the intensity of both 17 KDa and 14 KDa bands was significantly reduced in rcd1 at 7 wks but remained close to levels detected in normal retinas at 16 wks. In xlpra2, the intensity of the 17 KDa band was highly increased in 7 and 16 wks, while the 14 KDa band was decreased ~10 fold at 7 wks and remained close to normal at 16 wks. In erd, the 17 KDa band was upregulated at 8 and 12 wks but the intensity of the 14 KDa band remained close to normal. For comparison, the intensity of the 17 KDa band also was upregulated in xlpra1 at 16 wks, and the intensity of the 14 KDa band remained close to the level in normal retina. Finally, western blots with the acetyl-Histone H3 specific antibody showed clear upregulation of the acetylated histones H3 (detects a band of approximately 17 KDa (predicted molecular weight: 15 KDa)) at 16 wks in xlpra2, but no detectable changes in the acetylated H3 in other RD retinas (Fig 2B).

## Discussion

In this study we expand on our previous work [37] that examined the role of pro-apoptotic pathways in three early-onset canine models of retinal degeneration (rcd1, xlpra2 and erd). Here we focus on the broad nature of immune response along the time line of retinal degeneration (RD) in the same three early-onset models as well as in late-onset xlpra1. To characterize immune response in these four RD models, we have analyzed expression of a subset of key pro-inflammatory and anti-inflammatory/neuroprotective genes (Fig 1, S1 Table). We have also examined the protein expression of these genes as well as the potential activation of the inflammasome multiprotein complex.

In the early-onset models we found an upregulation of pro-inflammatory genes already begins at the *induction* and *execution* phases of RD. These genes include NLRP3 inflammasome components (*NLRP3*, *PYCARD* and *CASP1*), as well as the caspase-1-dependent pro-inflammatory cytokines (*IL1B* and *IL18*) and their receptors. Expression of common components of IL1B-, IL18- and TLR4- signaling pathways, e.g. *MYD88*, *IRAK4* and *TRAF6*, are also elevated. The observed upregulation takes place at various time points specific to the RD model, and it is in line with the morphological changes and time course of the photoreceptor cell death [33,37,45,46].

As expected for highly dynamic processes, gene expression and protein levels do not always correlate. Indeed, gene expression in immune cells is tightly regulated at both transcriptional and post-transcriptional levels to insure both rapid induction of the immune response and its timely shutdown to avoid excessive tissue damage. This notion has been highlighted in a recent study of translational regulation in the early phase of the macrophage response that showed quick translational activation of many feedback inhibitors of the inflammatory response, including NF-kB, a p38 MAPK antagonist and post-transcriptional suppressors of cytokine expression [63]. Therefore, we also focused on the protein levels of several pro-inflammatory and anti-inflammatory/neuroprotective genes and also determined the cell types in which they were expressed.

The secretion of pro-inflammatory IL1B and IL18 is an important part of the innate immune response [64–66] which, if left unregulated, may cause damage to host tissues and even cause host-cell death. Therefore, protein expression and secretion of these cytokines are tightly regulated to ensure that potent inflammatory response occurs only in the presence of a *bona fide* stimulus [67–69]. To evaluate putative roles of IL1B and IL18 pathways in the disease progression we examined production of mature IL1B and IL18 in retinal protein extracts in early-onset diseases by western blot analysis. While we were not able to detect mature IL18 in any of the models, we observed an increase in mature IL1B levels in rcd1 at 16 wks and in xlpra2 at 7 wks, an indication of caspase-1 activation during disease progression. These findings point to different mechanisms that control production and secretion of IL18 and IL1B cytokines in diseased retinas, and implicate importance of the IL1B pathway in the early-phase neuro-inflammatory responses in rcd1 and xlpra2 retinas.

Our results suggests that there is a bias toward the inflammatory M1 phenotype inside mixed microglia/macrophage population in early-onset diseases, particularly in rcd1 and xlpra2. This suggestion is indirectly supported by our previous results that showed upregulation of miR-155 in these diseases [70] as this microRNA is known to play an essential role in driving the M1 inflammatory phenotype [71]. Notably, double staining with PYCARD and CD18 identified microglia and infiltrating macrophages as the source of the inflammasome expression suggesting that the degenerating visual cells activate the inflammasome resulting in IL1B secretion and enhancing the degenerative process. In addition, activated IBA1^+^ microglia/macrophages invaded outer retinal layers in all diseased rcd1 and xlpra2 retinas (Figs 4 and 5). As our data indicate that microglia express inflammasome components, we postulate that damage-associated molecular patterns released during dysregulated retinal homeostasis initiate and perpetuate retinal glial cells response that further drives disease progression. Together, our results are consistent with published data indicating that secretion of IL1B by microglia in rd10 mice facilitates photoreceptor cell death [16].

In contrast to early-onset disease models, in late-onset xlpra1, we observed initiation of the immune response as early as at 16 wks, long before the time when the earliest structural changes and photoreceptor degeneration become apparent [36]. Such finding was not unexpected as we previously have found significant upregulation of GFAP transcript and protein levels in the same retinas, suggesting a disturbance in retinal homeostasis in the early phases of the disease [43]. It is possible that the early rod opsin mislocalization [44] induces release of inflammatory mediators that serve as an early initiation signal to the immune system. Indeed, we have identified early reactivity of retinal microglia in pre-degenerate retinas, supported by microglia redistribution from inner toward outer retinal layers (S1 Fig). However, dramatic increase in expression of *IL1B* and *TLR4* in xlpra1 in pre-degenerate retinas was not accompanied by upregulation of neither *IL18* nor any other components of IL1B and IL18 pathways. Future studies will be directed at identifying still other key genes and pathways to clarify the pathogenesis of xlpra1.

Inflammation, as a physiological response to tissue injury, should be resolved in a timely manner through the induction of endogenous anti-inflammatory mediators to minimize its deleterious consequences. In the present study, we examined the potential resolution of inflammation in early-onset diseases by analyzing the expression of 21 genes involved in anti-inflammatory and neuroprotective response. We found that the expression of most of these genes is similar to normal in 3 wks, 5 wks and 7 wks in both rcd1and xlpra2 models as well as in erd at 9.6–12 wks retinas.

We then focused on a subset of genes capable of moderating microglia/macrophage-mediated immune response, such as *IL4*, *IL13*, *CX3CL1*, *MANF*, *TGFB1* and *TGFB2*. A similar increase in *TGFB1* expression was present in rcd1 and xlpra2 at 16 wks, and in erd at 9.6–12 wks. Also, *IL4* was slightly upregulated in rcd1 at 7 and 16 wks, and *CX3CL1* was increased in rcd1 at 16 wks. However, upregulated gene expression of these moderating cytokines, did not appear to be sufficient to counteract expression of the pro-inflammatory genes (this study and [37]), and was not able to prevent apoptotic death of photoreceptors (previous study [37]), although it may have played a role in moderating early onset disease process.

In contrast, the early rise in expression of *IL4* and *FGF2* in the pre-diseased xlpra1 retinas may manifest more efficient counterbalancing act between pro- and anti-inflammatory cytokines potentially responsible for the slow disease progression and prolonged visual cell survival in xlpra1.

Lastly, we examined the expression of a subset of genes encoding HDACs and HATs in a pilot analysis to determine if changes in the level of histone acetylation in canine retinal disease models are associated with altered expression of immune response genes. Histone acetylation and deacetylation play critical roles in the control of pro-inflammatory gene transcription by regulating the access of transcription factors to pro-inflammatory gene promoters. Disruption of the acetylation/deacetylation balance may lead to sustained transcription of pro-inflammatory genes controlled by NF-kB and AP-1, resulting in increased influx of activated microglia/macrophages to already inflamed tissue and potentially creating the chronic cycle of inflammation that is the hallmark of neurodegenerative diseases [62]. While we detected only a mild decrease in expression of a small subset of HDAC genes in early-onset xlpra2 and erd, we have observed variable intensity of 17 KDa and 14 KDa bands that correspond to the molecular weight of histones in all four disease models. Western blot analysis with acetylated-Lysine antibody shows a misbalance in total histone acetylation level that is accompanied by expression changes of in immune response genes. Future studies will address the physiological significance of this important observation.

In summary, analyses of the inflammatory response in the current study allows us to re-interpret conclusions drawn based on characterization of pro-apoptotic pathways in our previous study [37]. Fast developing host immune response in rcd1 and xlpra2, as evaluated by upregulation of expression of pro-inflammatory genes, intraretinal migration and activation of microglia as well as secretion of mature IL1B, indicate timely shift from apoptotic to necroptotic and/or caspase-1 dependent cell death pathways for degenerating photoreceptors. In contrast, in erd, a fairly strong pro-inflammatory response at the peak of photoreceptors death was not accompanied by neither increase in intraretinal migration and activation of microglia nor by secretion of mature IL1B pointing out to possibility of secondary necrosis of apoptotic photoreceptors due to deficiency in clearance of apoptotic cells by resident microglia.

In comparison to early onset models, in late-onset xlpra1 the pro-inflammatory genes activated long before development of the disease phenotype are promptly counterbalanced by an adequate anti-inflammatory response. An intricate balance between pro- and anti-inflammatory factors may be the underlying cause of delayed progression in xlpra1 disease. However further studies are required to clarify the exact mechanism underlying progression of retinal degeneration in xlpra1.

Our studies further suggest that anti-inflammatory therapeutic intervention may be considered to moderate the inflammatory process that accompanies some of the inherited retinal degenerations.

## Supporting information

S1 Fig Double immunolabeling with CD18 and IBA1antibodies in rcd1 and xlpra2 at 3 wks (A1-A3) and at 5 wks (B1-B3). Immunolabeling of younger (3 wks, 5wks) normal and disease (rcd1, xlpra2) retina was done using CD18 (green) and IBA1 (red) antibodies. CD18 and IBA1 labeling is evident in normal, rcd1 and xlpra2 at all ages studied however labeling is more prominent in disease retinas, especially as disease progresses. CD18 and IBA1 co-localize in both rcd1 (A2, B2) and xlpra2 (A3, B3), yet, distribution of labeling varies among ages and diseases. At 3 wks age, prior to the reported peak of cell death for rcd1 (5 wks) and xlpra2 (6 wks), redistribution of immunolabeled cells towards upper layers is evident, nevertheless, intensity is higher in rcd1 (A2), whereas number of migrating cells in upper layers is higher in xlpra2. Furthermore, the distribution of 3 subpopulations of immunolabeled cells differs between diseases. In rcd1 most CD18^+^/IBA1^+^ cells (yellow) is located in IPL and GCL (A2) whereas xlpra2 shows CD18^+^/IBA1^+^ in the OPL as well (A3). Interestingly, CD18^-^/IBA1^+^ cells (red) are more prominent in OPL and INL (arrow) of xlpra2 (A3). At the peak of cell death (5 wks), CD18^+^ and IBA1^+^ cells number is more abundant than at 3wks of age, and they are present in IPL, INL and OPL (B2 and B3). At 5 wks, CD18^-^/IBA1^+^ cells (red) are present in OPL and INL in both diseases. Notes: ONL = outer nuclear layer; OPL = outer plexiform layer; INL = inner nuclear layer; GCL = ganglion cells layer. Scale bar 40 μm.
(TIF)

S2 Fig Immunohistochemical analysis of xlpra1 pre-degenerate carrier retina. Immunolabeling of normal and pre-degenerate carrier (xlpra1) retinas was done using rod opsin, PYCARD and CD18 antibodies. Immunolabeling with microglia/macrophage marker CD18 (green) antibody demonstrate migration of CD18^+^ cells toward upper retinal layers in xlpra1 carrier (A2, B2) in comparison with normal retina (A1, B1). In contrast, PYCARD intensity in carrier xlpra1 (A2) remains similar to normal retina of similar age (A1), however, carrier xlpra1 contain increased CD18^+^/PYCARD^+^ cell density (A2). Double immunolabeling with CD18 (green) and rod opsin (red) antibodies shows an increased density of CD18^+^ cells in proximity to the patches of rod opsin delocalization (B2) in carrier xlpra1, which represent the mutant region in the retina (arrows). The delocalization is visualized best without DAPI. Notes: ONL = outer nuclear layer. Scale bar 40 μm.
(TIF)

S1 Table List of genes tested by qRT-PCR. Genes are divided into three groups: (1) pro-inflammatory immune response; (2) neuroprotective and anti-inflammatory; (3) histone deacetylases and histone acetyltransferases.
(DOCX)

S2 Table List of primary antibodies successfully used in the current study. (DOCX)

S3 Table List of primary antibodies that were tested but failed to detect by IHC or western blot the canine specific antigen. (DOCX)

S4 Table Non-differentially expressed genes in study models: Pro-inflammatory immune response group. Results show fold changes that did not reach statistical significance between rcd1, xlpra2, erd and xlpra1 mutants compared to normal at different ages.
(DOCX)

S5 Table Non-differentially expressed genes in study models: Neuroprotective and anti-inflammatory group. (DOCX)

S6 Table Comparative analysis of gene expression in study models: Histone deacetylases and histone acetyltransferases group. Differentially expressed genes (p<0.05 and FC≥+/-2) are marked in red.
(DOCX)

## References

[1] Eandi CM, Charles Messance H, Augustin S, Dominguez E, Lavalette S, Forster V, et al Subretinal mononuclear phagocytes induce cone segment loss via IL-1beta. Elife. 2016;5:10.7554/eLife.16490 PMC4969036 27438413

[2] Gupta N, Brown KE, Milam AH. Activated microglia in human retinitis pigmentosa, late-onset retinal degeneration, and age-related macular degeneration. Exp Eye Res. 2003;76: 463–471. 12634111 10.1016/s0014-4835(02)00332-9

[3] Whitcup SM, Nussenblatt RB, Lightman SL, Hollander DA. Inflammation in retinal disease. Int J Inflam. 2013;2013: 724648 10.1155/2013/724648 24109539 PMC3784265

[4] Yoshida N, Ikeda Y, Notomi S, Ishikawa K, Murakami Y, Hisatomi T, et al Clinical evidence of sustained chronic inflammatory reaction in retinitis pigmentosa. Ophthalmology. 2013;120: 100–105. 10.1016/j.ophtha.2012.07.006 22986109

[5] Rock KL, Kono H. The inflammatory response to cell death. Annu Rev Pathol. 2008;3: 99–126. 10.1146/annurev.pathmechdis.3.121806.151456 18039143 PMC3094097

[6] Pasparakis M, Vandenabeele P. Necroptosis and its role in inflammation. Nature. 2015;517: 311–320. 10.1038/nature14191 25592536

[7] Duewell P, Kono H, Rayner KJ, Sirois CM, Vladimer G, Bauernfeind FG, et al NLRP3 inflammasomes are required for atherogenesis and activated by cholesterol crystals. Nature. 2010;464: 1357–1361. 10.1038/nature08938 20428172 PMC2946640

[8] Gicquel T, Victoni T, Fautrel A, Robert S, Gleonnec F, Guezingar M, et al Involvement of purinergic receptors and NOD-like receptor-family protein 3-inflammasome pathway in the adenosine triphosphate-induced cytokine release from macrophages. Clin Exp Pharmacol Physiol. 2014;41: 279–286. 10.1111/1440-1681.12214 24472059

[9] Mariathasan S, Monack DM. Inflammasome adaptors and sensors: intracellular regulators of infection and inflammation. Nat Rev Immunol. 2007;7: 31–40. 10.1038/nri1997 17186029

[10] Robert S, Gicquel T, Victoni T, Valenca S, Barreto E, Bailly-Maitre B, et al Involvement of matrix metalloproteinases (MMPs) and inflammasome pathway in molecular mechanisms of fibrosis. Biosci Rep. 2016;36:10.1042/BSR20160107 PMC4945993 27247426

[11] Rajan JV, Warren SE, Miao EA, Aderem A. Activation of the NLRP3 inflammasome by intracellular poly I:C. FEBS Lett. 2010;584: 4627–4632. 10.1016/j.febslet.2010.10.036 20971108 PMC3005299

[12] Baroja-Mazo A, Martin-Sanchez F, Gomez AI, Martinez CM, Amores-Iniesta J, Compan V, et al The NLRP3 inflammasome is released as a particulate danger signal that amplifies the inflammatory response. Nat Immunol. 2014;15: 738–748. 10.1038/ni.2919 24952504

[13] Guo H, Callaway JB, Ting JP. Inflammasomes: mechanism of action, role in disease, and therapeutics. Nat Med. 2015;21: 677–687. 10.1038/nm.3893 26121197 PMC4519035

[14] Tarallo V, Hirano Y, Gelfand BD, Dridi S, Kerur N, Kim Y, et al DICER1 loss and Alu RNA induce age-related macular degeneration via the NLRP3 inflammasome and MyD88. Cell. 2012;149: 847–859. 10.1016/j.cell.2012.03.036 22541070 PMC3351582

[15] Kataoka K, Matsumoto H, Kaneko H, Notomi S, Takeuchi K, Sweigard JH, et al Macrophage- and RIP3-dependent inflammasome activation exacerbates retinal detachment-induced photoreceptor cell death. Cell Death Dis. 2015;6: e1731 10.1038/cddis.2015.73 25906154 PMC4650542

[16] Zhao L, Zabel MK, Wang X, Ma W, Shah P, Fariss RN, et al Microglial phagocytosis of living photoreceptors contributes to inherited retinal degeneration. EMBO Mol Med. 2015;7: 1179–1197. 10.15252/emmm.201505298 26139610 PMC4568951

[17] Walsh JG, Reinke SN, Mamik MK, McKenzie BA, Maingat F, Branton WG, et al Rapid inflammasome activation in microglia contributes to brain disease in HIV/AIDS. Retrovirology. 2014;11: 35 10.1186/1742-4690-11-35 24886384 PMC4038111

[18] Sivakumar V, Foulds WS, Luu CD, Ling EA, Kaur C. Retinal ganglion cell death is induced by microglia derived pro-inflammatory cytokines in the hypoxic neonatal retina. J Pathol. 2011;224: 245–260. 10.1002/path.2858 21404274

[19] Rivera JC, Sitaras N, Noueihed B, Hamel D, Madaan A, Zhou T, et al Microglia and interleukin-1beta in ischemic retinopathy elicit microvascular degeneration through neuronal semaphorin-3A. Arterioscler Thromb Vasc Biol. 2013;33: 1881–1891. 10.1161/ATVBAHA.113.301331 23766263

[20] Cherry JD, Olschowka JA, O'Banion MK. Neuroinflammation and M2 microglia: the good, the bad, and the inflamed. J Neuroinflammation. 2014;11: 98 10.1186/1742-2094-11-98 24889886 PMC4060849

[21] Cherry JD, Olschowka JA, O'Banion MK. Are "resting" microglia more "m2"? Front Immunol. 2014;5: 594 10.3389/fimmu.2014.00594 25477883 PMC4235363

[22] Mills CD, Kincaid K, Alt JM, Heilman MJ, Hill AM. M-1/M-2 macrophages and the Th1/Th2 paradigm. J Immunol. 2000;164: 6166–6173. 28923981 10.4049/jimmunol.1701141

[23] Gordon S, Martinez FO. Alternative activation of macrophages: mechanism and functions. Immunity. 2010;32: 593–604. 10.1016/j.immuni.2010.05.007 20510870

[24] Zhang B, Bailey WM, Braun KJ, Gensel JC. Age decreases macrophage IL-10 expression: Implications for functional recovery and tissue repair in spinal cord injury. Exp Neurol. 2015;273: 83–91. 10.1016/j.expneurol.2015.08.001 26263843 PMC4644435

[25] Zhang F, Wang H, Wang X, Jiang G, Liu H, Zhang G, et al TGF-beta induces M2-like macrophage polarization via SNAIL-mediated suppression of a pro-inflammatory phenotype. Oncotarget. 2016. 10.18632/oncotarget.10561 PMC5239552 27418133

[26] Gong D, Shi W, Yi SJ, Chen H, Groffen J, Heisterkamp N. TGFbeta signaling plays a critical role in promoting alternative macrophage activation. BMC Immunol. 2012;13: 31 10.1186/1471-2172-13-31 22703233 PMC3406960

[27] Neves J, Zhu J, Sousa-Victor P, Konjikusic M, Riley R, Chew S, et al Immune modulation by MANF promotes tissue repair and regenerative success in the retina. Science. 2016;353: aaf3646 10.1126/science.aaf3646 27365452 PMC5270511

[28] Zabel MK, Zhao L, Zhang Y, Gonzalez SR, Ma W, Wang X, et al Microglial phagocytosis and activation underlying photoreceptor degeneration is regulated by CX3CL1-CX3CR1 signaling in a mouse model of retinitis pigmentosa. Glia. 2016;64: 1479–1491. 10.1002/glia.23016 27314452 PMC4958518

[29] Wang M, Ma W, Zhao L, Fariss RN, Wong WT. Adaptive Muller cell responses to microglial activation mediate neuroprotection and coordinate inflammation in the retina. J Neuroinflammation. 2011;8: 173 10.1186/1742-2094-8-173 22152278 PMC3251543

[30] Wang M, Wong WT. Microglia-Muller cell interactions in the retina. Adv Exp Med Biol. 2014;801: 333–338. 10.1007/978-1-4614-3209-8_42 24664715 PMC4685688

[31] Suber ML, Pittler SJ, Qin N, Wright GC, Holcombe V, Lee RH, et al Irish setter dogs affected with rod/cone dysplasia contain a nonsense mutation in the rod cGMP phosphodiesterase beta-subunit gene. Proc Natl Acad Sci U S A. 1993;90: 3968–3972. 8387203 10.1073/pnas.90.9.3968 PMC46427

[32] Ray K, Baldwin VJ, Acland GM, Blanton SH, Aguirre GD. Cosegregation of codon 807 mutation of the canine rod cGMP phosphodiesterase beta gene and rcd1. Invest Ophthalmol Vis Sci. 1994;35: 4291–4299. 8002249

[33] Berta AI, Boesze-Battaglia K, Genini S, Goldstein O, O'Brien PJ, Szel A, et al Photoreceptor cell death, proliferation and formation of hybrid rod/S-cone photoreceptors in the degenerating STK38L mutant retina. PLoS One. 2011;6: e24074 10.1371/journal.pone.0024074 21980341 PMC3184085

[34] Goldstein O, Kukekova AV, Aguirre GD, Acland GM. Exonic SINE insertion in STK38L causes canine early retinal degeneration (erd). Genomics. 2010;96: 362–368. 10.1016/j.ygeno.2010.09.003 20887780 PMC2996878

[35] Zhang Q, Acland GM, Wu WX, Johnson JL, Pearce-Kelling S, Tulloch B, et al Different RPGR exon ORF15 mutations in Canids provide insights into photoreceptor cell degeneration. Hum Mol Genet. 2002;11: 993–1003. 11978759 10.1093/hmg/11.9.993

[36] Zeiss CJ, Acland GM, Aguirre GD. Retinal pathology of canine X-linked progressive retinal atrophy, the locus homologue of RP3. Invest Ophthalmol Vis Sci. 1999;40: 3292–3304. 10586956

[37] Genini S, Beltran WA, Aguirre GD. Up-regulation of tumor necrosis factor superfamily genes in early phases of photoreceptor degeneration. PLoS One. 2013;8: e85408 10.1371/journal.pone.0085408 24367709 PMC3868615

[38] Yabal M, Muller N, Adler H, Knies N, Gross CJ, Damgaard RB, et al XIAP restricts TNF- and RIP3-dependent cell death and inflammasome activation. Cell Rep. 2014;7: 1796–1808. 10.1016/j.celrep.2014.05.008 24882010

[39] Wicki S, Gurzeler U, Wei-Lynn Wong W, Jost PJ, Bachmann D, Kaufmann T. Loss of XIAP facilitates switch to TNFalpha-induced necroptosis in mouse neutrophils. Cell Death Dis. 2016;7: e2422 10.1038/cddis.2016.311 27735938 PMC5133978

[40] Lagali PS, Picketts DJ. Matters of life and death: the role of chromatin remodeling proteins in retinal neuron survival. J Ocul Biol Dis Infor. 2011;4: 111–120. 10.1007/s12177-012-9080-3 23289056 PMC3382293

[41] Nielsen HM, Tost J. Epigenetic changes in inflammatory and autoimmune diseases. Subcell Biochem. 2013;61: 455–478. 10.1007/978-94-007-4525-4_20 23150263

[42] Shanmugam MK, Sethi G. Role of epigenetics in inflammation-associated diseases. Subcell Biochem. 2013;61: 627–657. 10.1007/978-94-007-4525-4_27 23150270

[43] Appelbaum T, Becker D, Santana E, Aguirre GD. Molecular studies of phenotype variation in canine RPGR-XLPRA1. Mol Vis. 2016;22: 319–331. 27122963 PMC4830396

[44] Beltran WA, Acland GM, Aguirre GD. Age-dependent disease expression determines remodeling of the retinal mosaic in carriers of RPGR exon ORF15 mutations. Invest Ophthalmol Vis Sci. 2009;50: 3985–3995. 10.1167/iovs.08-3364 19255154 PMC2718058

[45] Beltran WA, Hammond P, Acland GM, Aguirre GD. A frameshift mutation in RPGR exon ORF15 causes photoreceptor degeneration and inner retina remodeling in a model of X-linked retinitis pigmentosa. Invest Ophthalmol Vis Sci. 2006;47: 1669–1681. 10.1167/iovs.05-0845 16565408

[46] Acland GM, Aguirre GD. Retinal degenerations in the dog: IV. Early retinal degeneration (erd) in Norwegian elkhounds. Exp Eye Res. 1987;44: 491–521. 3496233 10.1016/s0014-4835(87)80160-4

[47] Farber DB, Danciger JS, Aguirre G. The beta subunit of cyclic GMP phosphodiesterase mRNA is deficient in canine rod-cone dysplasia 1. Neuron. 1992;9: 349–356. 1323314 10.1016/0896-6273(92)90173-b

[48] Gardiner KL, Downs L, Berta-Antalics AI, Santana E, Aguirre GD, Genini S. Photoreceptor proliferation and dysregulation of cell cycle genes in early onset inherited retinal degenerations. BMC Genomics. 2016;17: 221 10.1186/s12864-016-2477-9 26969498 PMC4788844

[49] Bustin SA, Benes V, Garson JA, Hellemans J, Huggett J, Kubista M, et al The MIQE guidelines: minimum information for publication of quantitative real-time PCR experiments. Clin Chem. 2009;55: 611–622. 10.1373/clinchem.2008.112797 19246619

[50] Livak KJ, Schmittgen TD. Analysis of relative gene expression data using real-time quantitative PCR and the 2(-Delta Delta C(T)) Method. Methods. 2001;25: 402–408. 10.1006/meth.2001.1262 11846609

[51] Maier T, Guell M, Serrano L. Correlation of mRNA and protein in complex biological samples. FEBS Lett. 2009;583: 3966–3973. 10.1016/j.febslet.2009.10.036 19850042

[52] Carpenter S, Ricci EP, Mercier BC, Moore MJ, Fitzgerald KA. Post-transcriptional regulation of gene expression in innate immunity. Nat Rev Immunol. 2014;14: 361–376. 10.1038/nri3682 24854588

[53] Kikkawa S, Matsumoto M, Shida K, Fukumori Y, Toyoshima K, Seya T. Human macrophages produce dimeric forms of IL-18 which can be detected with monoclonal antibodies specific for inactive IL-18. Biochem Biophys Res Commun. 2001;281: 461–467. 10.1006/bbrc.2001.4378 11181070

[54] Sasaki Y, Ohsawa K, Kanazawa H, Kohsaka S, Imai Y. Iba1 is an actin-cross-linking protein in macrophages/microglia. Biochem Biophys Res Commun. 2001;286: 292–297. 10.1006/bbrc.2001.5388 11500035

[55] Guyon R, Pearce-Kelling SE, Zeiss CJ, Acland GM, Aguirre GD. Analysis of six candidate genes as potential modifiers of disease expression in canine XLPRA1, a model for human X-linked retinitis pigmentosa 3. Mol Vis. 2007;13: 1094–1105. 17653054 PMC2779147

[56] Provis JM, Diaz CM, Penfold PL. Microglia in human retina: a heterogeneous population with distinct ontogenies. Perspect Dev Neurobiol. 1996;3: 213–222. 8931095

[57] Noailles A, Fernandez-Sanchez L, Lax P, Cuenca N. Microglia activation in a model of retinal degeneration and TUDCA neuroprotective effects. J Neuroinflammation. 2014;11: 186 10.1186/s12974-014-0186-3 25359524 PMC4221719

[58] Fontaine V, Kinkl N, Sahel J, Dreyfus H, Hicks D. Survival of purified rat photoreceptors in vitro is stimulated directly by fibroblast growth factor-2. J Neurosci. 1998;18: 9662–9672. 9822727 10.1523/JNEUROSCI.18-23-09662.1998 PMC6793308

[59] Traverso V, Kinkl N, Grimm L, Sahel J, Hicks D. Basic fibroblast and epidermal growth factors stimulate survival in adult porcine photoreceptor cell cultures. Invest Ophthalmol Vis Sci. 2003;44: 4550–4558. 14507904 10.1167/iovs.03-0460

[60] Filgueiras LR, Brandt SL, Ramalho TR, Jancar S, Serezani CH. Imbalance between HDAC and HAT activities drives aberrant STAT1/MyD88 expression in macrophages from type 1 diabetic mice. J Diabetes Complications. 2016. 10.1016/j.jdiacomp.2016.08.001 PMC5296405 27623388

[61] Cantley MD, Haynes DR. Epigenetic regulation of inflammation: progressing from broad acting histone deacetylase (HDAC) inhibitors to targeting specific HDACs. Inflammopharmacology. 2013;21: 301–307. 10.1007/s10787-012-0166-0 23341163

[62] Villagra A, Sotomayor EM, Seto E. Histone deacetylases and the immunological network: implications in cancer and inflammation. Oncogene. 2010;29: 157–173. 10.1038/onc.2009.334 19855430

[63] Schott J, Reitter S, Philipp J, Haneke K, Schafer H, Stoecklin G. Translational regulation of specific mRNAs controls feedback inhibition and survival during macrophage activation. PLoS Genet. 2014;10: e1004368 10.1371/journal.pgen.1004368 24945926 PMC4063670

[64] Weber A, Wasiliew P, Kracht M. Interleukin-1 (IL-1) pathway. Sci Signal. 2010;3: cm1. F 10.1126/scisignal.3105cm1 20086235

[65] Weinstock JV, Blum A, Metwali A, Elliott D, Arsenescu R. IL-18 and IL-12 signal through the NF-kappa B pathway to induce NK-1R expression on T cells. J Immunol. 2003;170: 5003–5007. 12734344 10.4049/jimmunol.170.10.5003

[66] Adachi O, Kawai T, Takeda K, Matsumoto M, Tsutsui H, Sakagami M, et al Targeted disruption of the MyD88 gene results in loss of IL-1- and IL-18-mediated function. Immunity. 1998;9: 143–150. 9697844 10.1016/s1074-7613(00)80596-8

[67] Lamkanfi M, Denecker G, Kalai M, D'hondt K, Meeus A, Declercq W, et al INCA, a novel human caspase recruitment domain protein that inhibits interleukin-1beta generation. J Biol Chem. 2004;279: 51729–51738. 10.1074/jbc.M407891200 15383541

[68] Munoz M, Eidenschenk C, Ota N, Wong K, Lohmann U, Kuhl AA, et al Interleukin-22 induces interleukin-18 expression from epithelial cells during intestinal infection. Immunity. 2015;42: 321–331. 10.1016/j.immuni.2015.01.011 25680273

[69] Radwan M, Stiefvater R, Grunert T, Sharif O, Miller I, Marchetti-Deschmann M, et al Tyrosine kinase 2 controls IL-1ss production at the translational level. J Immunol. 2010;185: 3544–3553. 10.4049/jimmunol.0904000 20713887 PMC2990881

[70] Genini S, Guziewicz KE, Beltran WA, Aguirre GD. Altered miRNA expression in canine retinas during normal development and in models of retinal degeneration. BMC Genomics. 2014;15: 172 10.1186/1471-2164-15-172 24581223 PMC4029133

[71] Jablonski KA, Gaudet AD, Amici SA, Popovich PG, Guerau-de-Arellano M. Control of the Inflammatory Macrophage Transcriptional Signature by miR-155. PLoS One. 2016;11: e0159724 10.1371/journal.pone.0159724 27447824 PMC4957803

