# Supplementary figures and images for: Strong upregulation of inflammatory genes accompanies photoreceptor demise in canine models of retinal degeneration
Source: PLoS One. 2017 May 9;12(5):e0177224. doi: 10.1371/journal.pone.0177224 (PMC5423635; doi:10.1371/journal.pone.0177224)

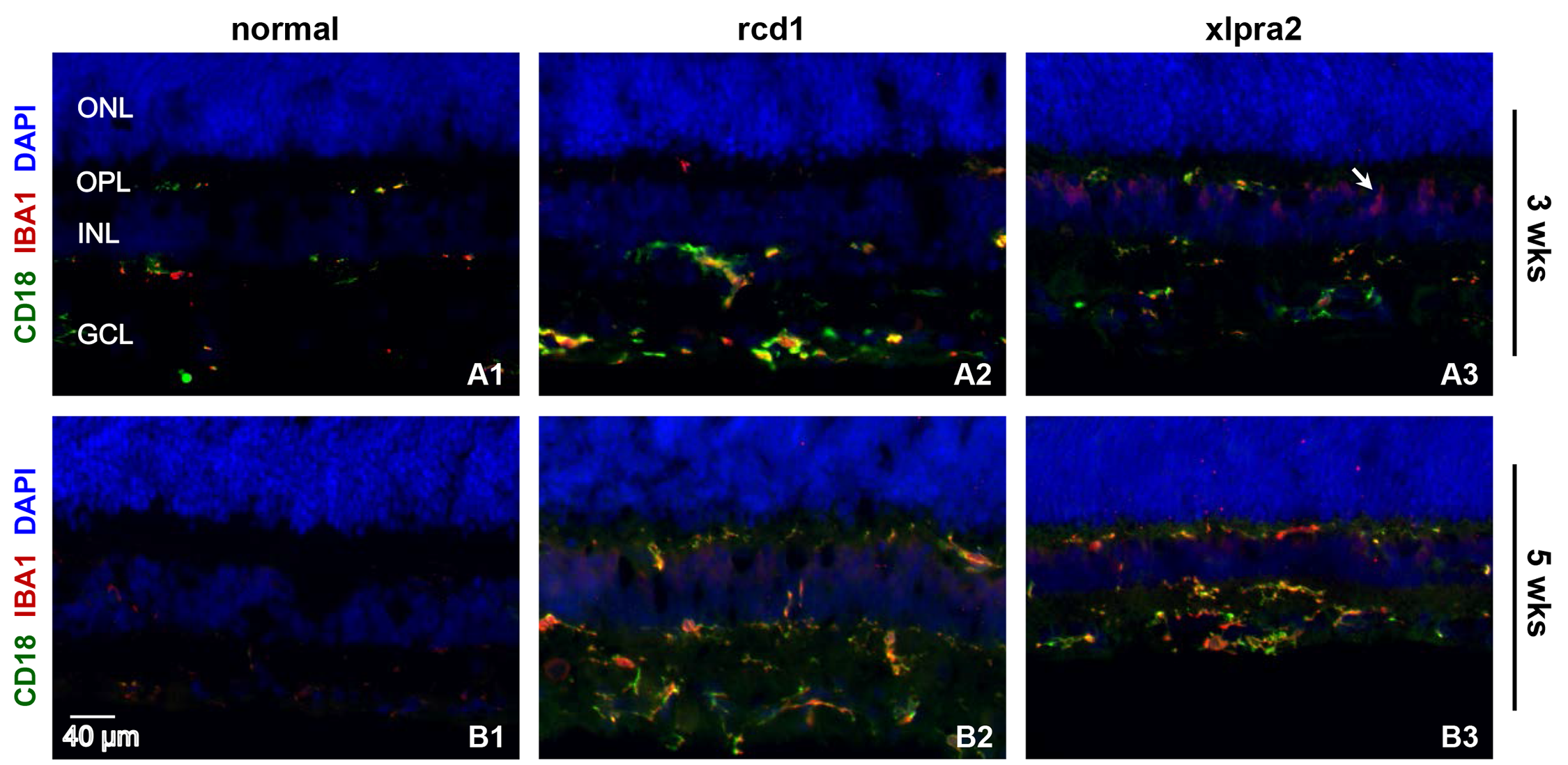

Supplement: S1 Fig — Immunolabeling of younger (3 wks, 5wks) normal and disease (rcd1, xlpra2) retina was done using CD18 (green) and IBA1 (red) antibodies. CD18 and IBA1 labeling is evident in normal, rcd1 and xlpra2 at all ages studied however labeling is more prominent in disease retinas, especially as disease progresses. CD18 and IBA1 co-localize in both rcd1 (A2, B2) and xlpra2 (A3, B3), yet, distribution of labeling varies among ages and diseases. At 3 wks age, prior to the reported peak of cell death for rcd1 (5 wks) and xlpra2 (6 wks), redistribution of immunolabeled cells towards upper layers is evident, nevertheless, intensity is higher in rcd1 (A2), whereas number of migrating cells in upper layers is higher in xlpra2. Furthermore, the distribution of 3 subpopulations of immunolabeled cells differs between diseases. In rcd1 most CD18+/IBA1+ cells (yellow) is located in IPL and GCL (A2) whereas xlpra2 shows CD18+/IBA1+ in the OPL as well (A3). Interestingly, CD18-/IBA1+ cells (red) are more prominent in OPL and INL (arrow) of xlpra2 (A3). At the peak of cell death (5 wks), CD18+ and IBA1+ cells number is more abundant than at 3wks of age, and they are present in IPL, INL and OPL (B2 and B3). At 5 wks, CD18-/IBA1+ cells (red) are present in OPL and INL in both diseases. Notes: ONL = outer nuclear layer; OPL = outer plexiform layer; INL = inner nuclear layer; GCL = ganglion cells layer. Scale bar 40 μm. (TIF) [file pone.0177224.s001.tif]

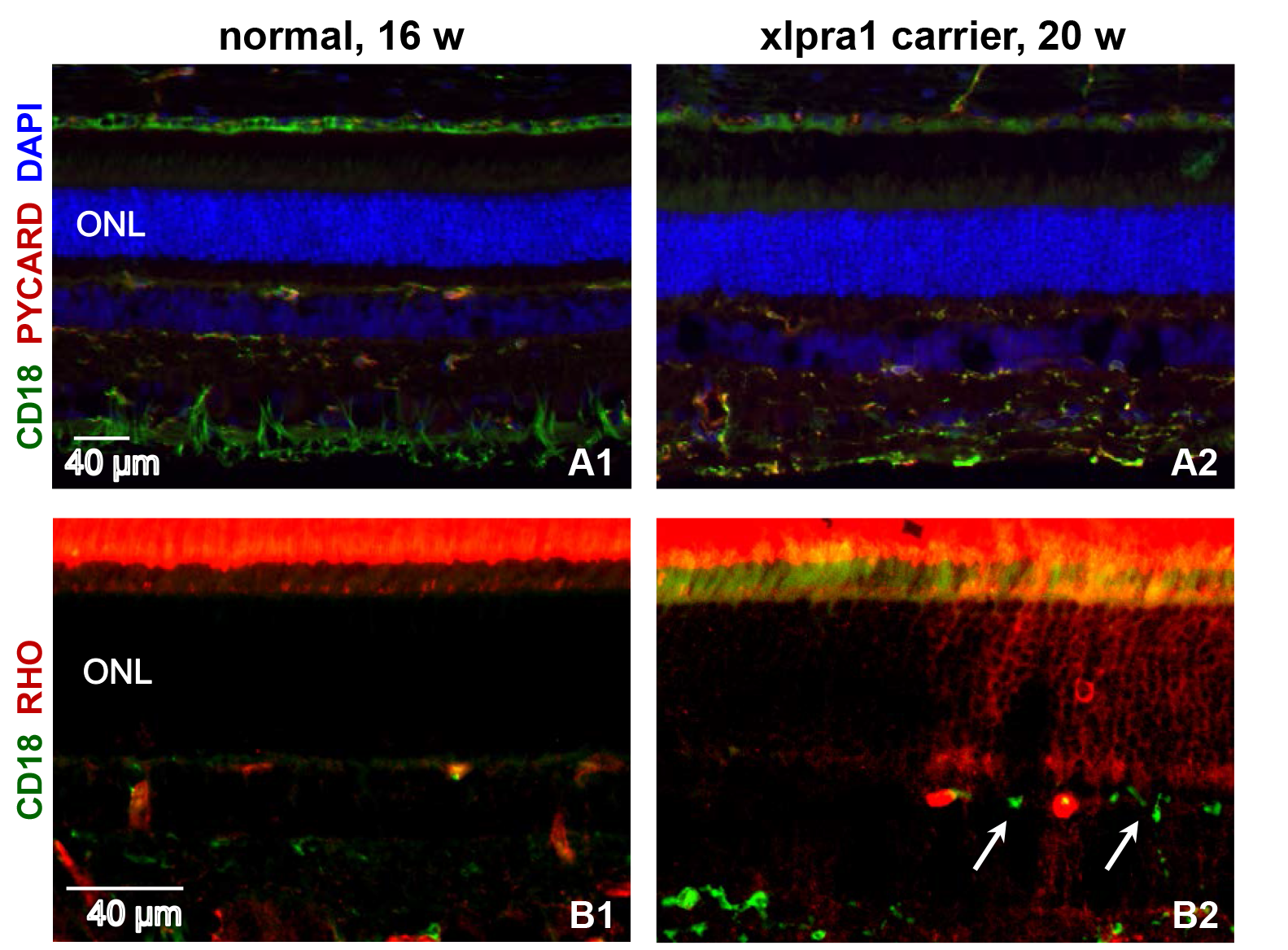

Supplement: S2 Fig — Immunolabeling of normal and pre-degenerate carrier (xlpra1) retinas was done using rod opsin, PYCARD and CD18 antibodies. Immunolabeling with microglia/macrophage marker CD18 (green) antibody demonstrate migration of CD18+ cells toward upper retinal layers in xlpra1 carrier (A2, B2) in comparison with normal retina (A1, B1). In contrast, PYCARD intensity in carrier xlpra1 (A2) remains similar to normal retina of similar age (A1), however, carrier xlpra1 contain increased CD18+/PYCARD+ cell density (A2). Double immunolabeling with CD18 (green) and rod opsin (red) antibodies shows an increased density of CD18+ cells in proximity to the patches of rod opsin delocalization (B2) in carrier xlpra1, which represent the mutant region in the retina (arrows). The delocalization is visualized best without DAPI. Notes: ONL = outer nuclear layer. Scale bar 40 μm. (TIF) [file pone.0177224.s002.tif]
