# Supplementary material for: Strong upregulation of inflammatory genes accompanies photoreceptor demise in canine models of retinal degeneration
Source: PLoS One. 2017 May 9;12(5):e0177224. doi: 10.1371/journal.pone.0177224 (PMC5423635; doi:10.1371/journal.pone.0177224)
Supplement: S1 Table — Genes are divided into three groups: (1) pro-inflammatory immune response; (2) neuroprotective and anti-inflammatory; (3) histone deacetylases and histone acetyltransferases. (DOCX) [file pone.0177224.s003.docx]

**S1 Table.** **List of genes tested by qRT-PCR.**

| **Gene symbol (alternative symbol)** | **Gene description [NCBI transcript accession number]** | **Protein function / Pathway** | **Group*** | **Primer sequences (5’→3’)** |
| --- | --- | --- | --- | --- |
| *NLRP3* | NLR family pyrin domain containing 3 [XM_005623149] | inflammasome component / | 1 | **F:** GAGGAGAAGGCATGGGCCATG  **R:** CCAATAAACCCAACCACTCCTCTTCAA |
| *CASP1* | caspase 1 [EU183118] | inflammasome component / | 1 | **F:** TGGAGCTGAACTTGACATTGCAGG  **R:** AATTCCCGTAGCACTGATTCCATACC |
| *PYCARD (ASC)* | PYD and CARD domain containing [DN347564] | inflammasome component / | 1 | **F:** GACCTCACACAAAGGCCACACTCC  **R:** CCCGGTGCTGGTCCACAAAG |
| *P2RX7* | purinergic receptor P2X7 [NM_001113456] | / involved in NLRP3 inflammasome activation | 1 | **F:** TGGAAGAGCTGTGCTGTCGGAAA  **R:** AGAGCAGGAGGAACTGCAGGACC |
| *SYK* | spleen associated tyrosine kinase [XM_005615953] | / involved in NLRP3 inflammasome activation | 1 | **F:** GATTTCGGACTTTCCAAAGCACTCC  **R:** GCGTACCACTTCACGGGCCAC |
| *IL1B* | interleukin 1 beta [NM_001037971] | inflammasome substrate / | 1 | **F:** GGAAATGTGAAGTGCTGCTGCCAA  **R:** GCAGGGCTTCTTCAGCTTCTCCAA |
| *IL1R1* | interleukin 1 receptor type I [DN334399] | / IL1B pathway | 1 | **F:** ATGAACCTAACTTCTGTTACAACACAGAAGC  **R:** CCCATTATGCGCCGTAGTCACATT |
| *IL1R2* | interleukin 1 receptor type 2 [DN364712] | / IL1B pathway | 1 | **F:** CCCATAACAAAATGGACATGAAGATTCAG  **R:** GTAAGCGAGAGGTTCCCTTCACATTTAGA |
| *IL18* | interleukin 18 [NM_001003169] | inflammasome substrate / | 1 | **F:** TTCTGACTGTACAGATAATGCACCCCA  **R:** ATACTATCCGGAGGACTCATTTTCTGAAAG |
| *IL18R1* | interleukin 18 receptor 1 [XM_014117427] | / IL18 pathway | 1 | **F:** TTGACTTGGCCCTATTTTATAGACATTGCA  **R:** CGGGTCCACATTCTCTTAGGAAAGACAC |
| *CXCL8 (IL8)* | C-X-C motif chemokine ligand 8 [NM_001003200] | a chemokine produced by immune cells / | 1 | **F:** GACAGTGGCCCACAATGTGAAAACTC  **R:** GTTGTTTCACGGATCTTGTTTCTCAGC |
| *PTGES* | prostaglandin E synthase [NM_001122854] | production of prostaglandin E / IL1B, TNF, IFNγ pathways | 1 | **F:** TCGGAAGAAGGCTTTTGCCAACC  **R:** AAGGAGTAAACGAAGCCCAGGAACA |
| *TLR4* | toll like receptor 4 [NM_001002950] | / TLR4 stimulation controls NLRP3 Inflammasome activation | 1 | **F:** TTGCATGCAGGTGGTTGCTAACAC  **R:** CTCAGGCGGTTAAAGCTCAGGTCC |
| *MYD88* | myeloid differentiation primary response 88 [XM_534223] | / common adaptor protein in IL1B, IL18 and TLR4 pathways | 1 | **F:** GCATCACCATGCTTGATGATCCC  **R:** CTTCAGCCGATAGTTTGTCTGTTCCA |
| *IRAK4* | interleukin 1 receptor associated kinase 4 [XM_005636955] | / common adaptor protein in IL1B, IL18 and TLR4 pathways | 1 | **F:** CAAGATTGCCCAAGGTGCAGCTAA  **R:** CGGGCAAGCCCAAAGTCAGATA |
| *TRAF6* | TNF receptor associated factor 6 [XM_003432322] | / common adaptor protein in IL1B, IL18 and TLR4 pathways | 1 | **F:** GTCCAAATGAAGGTTGTCTGCACAAA  **R:** TTGGCACTGGAGACAATTCATGAGA |
| *CSF1R* | colony stimulating factor 1 receptor [DN426752] | / regulation of survival, proliferation and differentiation of mononuclear phagocytes | 1 | **F:** TACAAATATAAGCAGAAGCCCAAGTACCA  **R:** CGGCTCCGAGAGTCTTCCCAAAC |
| *CD200R* | CD200 receptor 1 [DN382519] | / control of microglia activation | 1 | **F:** CACCAAGTCCACAGCTCCTTTCATACC  **R:** CTCCTGTACCTATGCACTCAAACACACA |
| *CD74* | CD74 molecule [DN270150] | / microglial activation | 1 | **F:** ACACAGGACCACGTGATGCACC  **R:** GATACATCCAGTTCTCAAAGACCTTCCAA |
| *TXNIP* | thioredoxin interacting protein [DN426405] | / IL1B maturation pathway | 1 | **F:** GCTTCTCCTGGAAGACCAGCCAA  **R:** CAAGAAAAGCCTTCACCCAGTAGTCTACA |
| *VEGFA* | vascular endothelial growth factor A [NM_001003175] | / angiogenesis, vasculogenesis and endothelial cell growth | 1 | **F:** CCACCATGCCAAGTGGTCCCA  **R:** TGGAAGATCTCCACCACGGTCTCAA |
| *FLT1 (VEGFR1)* | fms related tyrosine kinase 1 [DN745772] | / VEGFA pathway | 1 | **F:** GATTGAGAGCATCACTCAGCGCAC  **R:** AGGTCCTCACCCTCCGTAGGCA |
| *KDR (VEGFR2)* | kinase insert domain receptor [NM_001048024] | / VEGFA pathway | 1 | **F:** TGGTTGTGAATGTCCCGCCC  **R:** CGCACTCCTCCAGCTGCCAG |
| *IL6* | interleukin 6 [NM001003301] | a pro-inflammatory cytokine / | 1 | **F:** GCACTGAGAAAGGAGATGTGTGACAAG  **R:** CCTGATTGAACCCAGATTGGAAGC |
| *TNF* | tumor necrosis factor [NM_001003244] | a pro-inflammatory cytokine / | 1 | **F:** CCCAGAGGGAAGAGCTCCCAAA  **R:** GGCTTGTCACTTGGGCTTCGAGAA |
| *IL18BP* | interleukin 18 binding protein [NM_001048018] | an inhibitor of the proinflammatory cytokine, IL18 / | 2 | **F:** GTCACCTGGCTGGAGCCACAC  **R:** AGACCGGCTGCAGGCAGTACA |
| *IL1RN* | interleukin 1 receptor antagonist [NM_001003096] | an inhibitor of the proinflammatory cytokine, IL1B / | 2 | **F:** TAACCAACTAGTCGCTGGATAGTTGCAA  **R:** CCCCCATGGATCCCCAAGAA |
| *CX3CL1 (fractalkin)* | C-X3-C motif chemokine ligand 1 [NM_001284456] | / control of microglial phagocytosis and activation | 2 | **F:** TGCTGGCCGGACAGCACCTC  **R:** CTTCAAGATGATGGCAGGCTTCCC |
| *CX3CR1* | C-X3-C motif chemokine receptor 1 [NM_001284491] | / control of microglial phagocytosis and activation | 2 | **F:** GACACATCAGACGTTCCCTTCCCAG  **R:** TGTCCCACAAATCACAGGCTTCA |
| *PDGFA* | platelet derived growth factor subunit A [NM_001190172] | / role in developmental processes in retina | 2 | **F:** GGAAAAGAAGCATTGAGCAGGCCA  **R:** CGCACAGCGCTTCACCTCCAC |
| *PDGFB* | platelet derived growth factor subunit B [NM_001003383] | / role in developmental processes in retina | 2 | **F:** CGGAGCCTCCGTAGACGAAGACG  **R:** AGGCTCCTTCTCCCTCGGGATAAA |
| *PDGFRA* | platelet derived growth factor receptor alpha [AY525124] | / PDGFA pathway | 2 | **F:** CTGGAGAGGTGAAAGGCAAAGGCA  **R:** TGGCGGGCAGCACATTCATAA |
| *PDGFRB* | platelet derived growth factor receptor beta [NM_001003382] | / PDGFB pathway | 2 | **F:** CAGCCTCCAGGTGTCATCCATCAA  **R:** CACTCTCCATGCGTGGGTATGTCC |
| *FGF2* | fibroblast growth factor 2 [AF060562] | a neurotrophic factor / | 2 | **F:** CGATCCCCACGTCAAATTGCAA  **R:** AATCGTTCAAAAAAGAAGCACTCGTCA |
| *FGFR1* | fibroblast growth factor receptor 1 [DN876573] | / FGF2 pathway | 2 | **F:** TCGAGGCTATAAGGTCCGTTACGCC  **R:** GGACCGCTCCACGACATCGA |
| *CNTFR* | ciliary neurotrophic factor receptor [NM_001003353] | / CNTFR pathway | 2 | **F:** CAGTCCTCAGGAAGCACCCCAC  **R:** GGTCTGTCCCATTTACCCGCC |
| *LIF* | leukemia inhibitory factor [NM_001197073] | a neurotrophic factor / | 2 | **F:** CACGCCACCCATGTCACAGC  **R:** TGGCGTGGAATGGTGGGAAG |
| *MANF* | mesencephalic astrocyte derived neurotrophic factor [GR896107] | a neurotrophic factor / | 2 | **F:** GATGCTGCCACTAAAATCATCAACGA  **R:** ACCCGGAGCTTCTTCAGGTCCAC |
| *TGFB1* | transforming growth factor beta 1 [NM_001003309] | / cells proliferation, differentiation and growth | 2 | **F:** GTGAGGCAGTGGCTGACCCAT  **R:** TCGGCGGCTGGAACTGAACC |
| *TGFB2* | transforming growth factor beta 2 [DQ525400] | / cells proliferation, differentiation and growth | 2 | **F:** TTCGTTTACAGAACCCGAAAGCCA  **R:** CTGCTCTCGTCTTGACGACTTTGC |
| *EGF* | epidermal growth factor [NM_001003094] | / cells proliferation and growth | 2 | **F:** CCTGCTTGTGTGGGTCCTGCAC  **R:** GATACACCAGCATCTGCCACCAATT |
| *EGFR* | epidermal growth factor receptor [AY527212] | / EGF pathway | 2 | **F:** GGCACAGTGTACAAGGGACTCTGGATC  **R:** GCCATCACGTAGGCTTCATCAAGAA |
| *IL4* | interleukin 4 [NM_001003159] | an anti-inflammatory cytokine / | 2 | **F:** ACTGCTCCAAAGAACACAAGCGATAA  **R:** ATGCTGCTGAGGTTCCTGTAGAGTCC |
| *IL13* | interleukin 13 [NM_001003384] | an anti-inflammatory cytokine / | 2 | **F:** GGAGCTCATTGAGGAGCTGGTCAAC  **R:** TGGCGCTGCAGTCGGAGACA |
| *IL10* | interleukin 10 [NM_001003077] | an anti-inflammatory cytokine / | 2 | **F:** AACCACGACCCAGACATCAAGAACC  **R:** TCCACCGCCTTGCTCTTATTCTCAC |
| *NTRK2 (TrkB)* | neurotrophic receptor tyrosine kinase 2 [CF410871] | receptor for BDNF neurotrophic factor and neurotrophin-4 / | 2 | **F:** CCCGACCGCTTTAGTCAGCAAC  **R:** GCCAACCCAAATTATCCCGACGT |
| *NTRK3 (TrkC)* | neurotrophic receptor tyrosine kinase 3 [DN904172] | receptor for neurotrophin-3 / | 2 | **F:** ACAAGCCAGACACGTGTTTCAGAGA  **R:** CCCAGCCTACCAAGGTGACATCAA |
| *IGF1* | insulin like growth factor 1 [NM_001313855] | a neurotrophic factor / | 2 | **F:** AATTCATTTCCAGACTTTGCACTTCAGA  **R:** GGTAGAAGAGGTGCGAGGAGGACA |
| *HDAC1* | histone deacetylase 1 [DN264657] | negatively regulates transcription via chromatin remodeling / | 3 | **F:** GCGCAGACTCAGGGCACCAG  **R:** TCATACGGATTCGGTGAGGCTTCA |
| *HDAC2* | histone deacetylase 2 [CF412014] | negatively regulates transcription via chromatin remodeling / | 3 | **F:** TAATGTTGCTCGATGTAGGACGTACG  **R:** TATGCAGTTTGAAGTCCGGGCCA |
| *HDAC3* | histone deacetylase 3 [DN353869] | negatively regulates transcription via chromatin remodeling / | 3 | **F:** CCATGGTGATGGAGTTCAGGAAGC  **R:** TCATACATATCACCTGTGCCAGGAAAGA |
| *HDAC4* | histone deacetylase 4 [XM_014107665] | negatively regulates transcription via chromatin remodeling / | 3 | **F:** GTGGCCAGCACGGAAGTGAAG  **R:** GGGAGCTCTGGTCGAGCGAAC |
| *HDAC5* | histone deacetylase 5 [DN398124] | negatively regulates transcription via chromatin remodeling / | 3 | **F:** GGCATTGGCGTGGACAGTGACA  **R:** GGGCCGGATGATGGCAAATC |
| *HDAC6* | histone deacetylase 6 [DN376478] | negatively regulates transcription via chromatin remodeling / | 3 | **F:** AGCCAAGCAGCAGAGCAAGACCTAA  **R:** CGCTCAGGGCCCTCAGGAAA |
| *HDAC9* | histone deacetylase 9 [DN265083] | negatively regulates transcription via chromatin remodeling / | 3 | **F:** GGACTGGCCCCAGCTCACCAA  **R:** CAGGTTCATGGAATCTTCATGAATTAGAA |
| *SIRT1* | sirtuin 1 [DN343144] | NAD-dependent protein deacetylase / | 3 | **F:** CAGTTGCTGACCCGGTGAGGAA  **R:** TCTGAATATACATCAGCGCCATGAAA |
| *SIRT2* | sirtuin 2 [DQ104221] | NAD-dependent protein deacetylase / | 3 | **F:** GAGTACCCGCTCAGCTGGATGAAA  **R:** GATGAGGAGGTCCACCATCAGGAAG |
| *KAT2A (GCN5)* | lysine acetyltransferase 2A [DN875411] | positively regulates transcription via chromatin remodeling / | 3 | **F:** CCTATGCCGACGAGTACGCCA  **R:** GGGATTCAGCTCACACTCCATCAA |
| *EP300* | E1A binding protein p300 [DN441566] | positively regulates transcription via chromatin remodeling / | 3 | **F:** CCGTCCTAAATGCTTGAGGACTGCAG  **R:** GGTCAGGAGGATGGCAATGGAAGATA |
| *CREBBP (CBP)* | CREB binding protein [Y11308] | positively regulates transcription via chromatin remodeling / | 3 | **F:** TCCCCACAGACTTTGTGCTGCTAC  **R:** TCGTCACCCAGGCTAACATTCTCC |
| *TAF1* | TATA-box binding protein associated factor 1 [DN368535] | positively regulates transcription via chromatin remodeling / | 3 | **F:** TGTCAAGCACAAAGGAACAAAGCC  **R:** GTTGTCAATGGGAAAGATGGAGTACCAA |
| *TBP* | TATA-box binding protein [DN272641] | a housekeeping protein |  | **F:** ACGGGAGCCAAGAGTGAAGAACAG  **R:** TTCACATCACAGCTCCCCACCA |

*Genes are divided into three groups: (1) pro-inflammatory immune response; (2) neuroprotective and anti-inflammatory; (3) histone deacetylases and histone acetyltransferases.
