# Supplementary material for: Strong upregulation of inflammatory genes accompanies photoreceptor demise in canine models of retinal degeneration
Source: PLoS One. 2017 May 9;12(5):e0177224. doi: 10.1371/journal.pone.0177224 (PMC5423635; doi:10.1371/journal.pone.0177224)
Supplement: S2 Table — (DOCX) [file pone.0177224.s004.docx]

**S2 Table.** **List of primary antibodies successfully used in the current study.**

| **Antigen/(species)** | **Host** | **Source, Catalog No.** | | **Dilution** | **Application** |
| --- | --- | --- | --- | --- | --- |
| CD18 (CA16:3C10) | mouse | | kindly provided by Dr. P. Moore | 1:20 | IHC* |
| IBA1 | rabbit | | Wako, 019-19741 | 1:200 | IHC |
| PYCARD | rabbit | | Adipogen, AG-25B-0006 | 1:1000/1:500 | IHC/WB* |
| Caspase-1 | goat | | Santa Cruz Biotech, sc-1780 | 1:200 | WB |
| NLRP3 | goat | | Novus Biologicals, NB100-41104 | 1:300 | WB |
| CSFR1 | rabbit | | Santa Cruz Biotech, sc-692 | 1:200 | WB |
| IL1B | rabbit | | Abcam, ab34837 | 1:300 | WB |
| IL1R1 | rabbit | | Aviva, ARP63914_P050 | 1:300 | WB |
| IL18 | rabbit | | Abcam, ab34837 | 1:300 | WB |
| IL18R1 | rabbit | | Abcam, ab117432 | 1:300 | WB |
| IRAK4 | mouse | | Santa Cruz Biotech, sc-374349 | 1:500 | WB |
| MyD88 | rabbit | | Santa Cruz Biotech, sc-11356 | 1:200 | WB |
| TLR4 | rabbit | | Aviva, ARP59160 | 1:300 | WB |
| TLR4 | mouse | | Abcam, ab22048 | 1:200 | IHC |
| Acetyl-Histone H3 | rabbit | | Cell Signaling, 9649 | 1:200 | WB |
| Acetylated Lysine | rabbit | | Cell Signaling, 9441 | 1:500 | WB |
|  |  | |  |  |  |

Notes: IHC = immunohistochemistry, WB = western blot
