# Supplementary material for: Strong upregulation of inflammatory genes accompanies photoreceptor demise in canine models of retinal degeneration
Source: PLoS One. 2017 May 9;12(5):e0177224. doi: 10.1371/journal.pone.0177224 (PMC5423635; doi:10.1371/journal.pone.0177224)
Supplement: S3 Table — (DOCX) [file pone.0177224.s005.docx]

**S3 Table. List of primary antibodies that were tested but failed to detect by IHC or western blot the canine specific antigen.**

| **Antigen/(species)** | **Host** | **Source, Catalog No.** | | **Dilution** | **Application** |
| --- | --- | --- | --- | --- | --- |
| Acetyl-Histone H2A | rabbit | | Cell Signaling, 2576 | 1:200 | IHC/WB |
| Acetyl-Histone H2B | rabbit | | Cell Signaling, 12799 | 1:200 | IHC/WB |
| Histone H2A | rabbit | | Cell Signaling, 12349 | 1:200 | IHC/WB |
| Histone H2B | rabbit | | Cell Signaling, 12364 | 1:200 | IHC/WB |
| caspase-1 | goat | | Santa Cruz Biotech, sc-1780 | 1:100 | IHC |
| CD200R1 | rabbit | | LifeSpan BioSciences, LS-C135630 | 1:200 | IHC |
| CD74 | goat | | Santa Cruz Biotech, sc-5440 | 1:100 | IHC |
| CSF1R | rabbit | | Santa Cruz Biotech, sc-692 | 1:100 | IHC |
| IL1B | goat | | R&D Systems, AF3747 | 1:100/1:200 | IHC/WB |
| IL4 | mouse | | R&D Systems, MAB7541 | 1:50/1:200 | IHC/WB |
| IL6 | mouse | | R&D Systems, MAB16091 | 1:50/1:200 | IHC/WB |
| IL6, human | rabbit | | Abcam, AB6672 | 1:500 | IHC |
| IL18, canine | mouse | | R&D Systems, MAB29241 | 1:500/1:200 | IHC/WB |
| IL18, canine | goat | | R&D Systems, AF2924 | 1:200/1:300 | IHC/WB |
| LIF, human | mouse | | R&D Systems, MAB250 | 1:500 | IHC |
| LIF, murine | rat | | Novus, NBP2-27406 | 1:100/1:200 | IHC/WB |
| NLRP3, human | goat | | Novus, NB100-41104 | 1:200 | IHC/WB |
| NLRP3, murine | rabbit | | Cell Signaling, 15101 | 1:500/1:200 | IHC/WB |
| NLRP3, murine | mouse | | Adipogen Life Sciences, AG-20B-0014 | 1:100 | IHC |
| P2RX7R, human | rabbit | | Sigma Aldrich, HPA034967 | 1:200 | IHC |
| TLR4, murine | mouse | | Santa Cruz Biotech, sc-293072 | 1:200 | IHC/WB |
