# Supplementary material for: Strong upregulation of inflammatory genes accompanies photoreceptor demise in canine models of retinal degeneration
Source: PLoS One. 2017 May 9;12(5):e0177224. doi: 10.1371/journal.pone.0177224 (PMC5423635; doi:10.1371/journal.pone.0177224)
Supplement: S4 Table — Results show fold changes that did not reach statistical significance between rcd1, xlpra2, erd and xlpra1 mutants compared to normal at different ages. (DOCX) [file pone.0177224.s006.docx]

**S4 Table. Non-differentially expressed genes in study models: pro-inflammatory immune response group.**

| **Gene** | **FC rcd1**  **vs. normal** | | **FC xlpra2**  **vs. normal** | **FC erd**  **vs. normal** | **FC xlpra1**  **vs. normal** | | |
| --- | --- | --- | --- | --- | --- | --- | --- |
|  | ***3 wks*** | ***3 wks*** | |  | |  |  |
| *NLRP3* | 1.4 | 1.1 | |  | |  |  |
| *CASP1* | -1.1 | 1.2 | |  | |  |  |
| *PYCARD* | 1.5 | 1.6 | |  | |  |  |
| *IL1B* | 1.3 | 1.5 | |  | |  |  |
| *IL1R2* | 1.2 | 1.1 | |  | |  |  |
| *IL18R1* | -1.1 | -1.2 | |  | |  |  |
| *TLR4* | -1.1 | 1.3 | |  | |  |  |
| *MYD88* | 1.5 | 1.1 | |  | |  |  |
| *IRAK4* | 1.4 | 1.6 | |  | |  |  |
| *P2RX7* | 1.4 | 1.2 | |  | |  |  |
| *SYK* | -1.1 | 1.1 | |  | |  |  |
| *TRAF6* | 1.4 | -1.2 | |  | |  |  |
| *PTGES* | 1.2 | 1.7 | |  | |  |  |
| *CSF1R* | 1.4 | 1.2 | |  | |  |  |
| *CD200R* | 1.5 | 1.1 | |  | |  |  |
| *CD74* | 1.2 | 1.4 | |  | |  |  |
| *TXNIP* | 1.1 | -1.1 | |  | |  |  |
| *VEGFA* | -1.3 | -1.6 | |  | |  |  |
| *FLT1* | -1.1 | 1.1 | |  | |  |  |
| *KDR* | -1.2 | -1.6 | |  | |  |  |
|  | ***5 wks*** | ***5 wks*** | |  | |  |  |
| *NLRP3* | 1.7 | 1.2 | |  | |  |  |
| *CASP1* | 1.6 | 1.1 | |  | |  |  |
| *PYCARD* | 1.6 | 1.4 | |  | |  |  |
| *IL1B* | 1.8 | -1.1 | |  | |  |  |
| *IL18R1* | 1.3 | 1.2 | |  | |  |  |
| *TLR4* | 1.6 | 1.3 | |  | |  |  |
| *MYD88* | 1.2 | 1.1 | |  | |  |  |
| *IRAK4* | 1.5 | 1.3 | |  | |  |  |
| *TRAF6* | 1.2 | 1.1 | |  | |  |  |
| *P2RX7* | 1.5 | 1.2 | |  | |  |  |
| *SYK* | 1.2 | 1.1 | |  | |  |  |
| *PTGES* | 1.4 | -1.6 | |  | |  |  |
| *CSF1R* | 1.3 | 1.4 | |  | |  |  |
| *CD200R* | 1.5 | 1.1 | |  | |  |  |
| *CD74* | 1.5 | 1.2 | |  | |  |  |
| *TXNIP* | -1.1. | 1.2 | |  | |  |  |
| *VEGFA* | -1.3 | -1.3 | |  | |  |  |
| *FLT1* | -1.4 | 1.2 | |  | |  |  |
| *KDR* | -1.4 | -1.1 | |  | |  |  |
|  | ***7 wks*** | ***7 wks*** | |  | |  |  |
| *IL18R1* | 1.4 | 1.6 | |  | |  |  |
| *MYD88* | 1.7 | 1.3 | |  | |  |  |
| *SYK* | 1.4 | 1.1 | |  | |  |  |
| *PTGES* | 1.1 | -1.6 | |  | |  |  |
| *TRAF6* | 1.2 | 1.3 | |  | |  |  |
| *CD200R* | 1.7 | 1.4 | |  | |  |  |
| *TXNIP* | 1.5 | 1.2 | |  | |  |  |
| *VEGFA* | 1.6 | 1.0 | |  | |  |  |
| *FLT1* | 1.7 | 1.2 | |  | |  |  |
| *KDR* | -1.1 | -1.3 | |  | |  |  |
|  | ***16 wks*** | ***16 wks*** | | ***9.6-12 wks*** | | ***16 wks*** |  |
| *PTGES* | 1.8 | 1.1 | | -1.1 | | -1.2 |  |
| *TRAF6* | 1.8 | 1.4 | | 1.4 | | 1.6 |  |
| *TXNIP* | 1.6 | 1.4 | | -1.2 | | 1.1 |  |
| *VEGFA* | 1.2 | 1.0 | | -1.1 | | 1.4 |  |
| *FLT1* | 1.4 | 1.2 | | 1.0 | | -1.1 |  |
| *KDR* | 1.2 | -1.1 | | -1.2 | | 1.2 |  |
| *IL6* |  |  | |  | | 1.2 |  |
|  |  |  | |  | |  |  |
