# Supplementary material for: Strong upregulation of inflammatory genes accompanies photoreceptor demise in canine models of retinal degeneration
Source: PLoS One. 2017 May 9;12(5):e0177224. doi: 10.1371/journal.pone.0177224 (PMC5423635; doi:10.1371/journal.pone.0177224)
Supplement: S5 Table — (DOCX) [file pone.0177224.s007.docx]

**S5 Table. Non-differentially expressed genes in study models: neuroprotective and anti-inflammatory group.**

| **Genes** | **FC rcd1**  **vs. normal** | | **FC xlpra2**  **vs. normal** | **FC erd**  **vs. normal** | **FC xlpra1**  **vs. normal** | | |
| --- | --- | --- | --- | --- | --- | --- | --- |
|  | ***3 wks*** | ***3 wks*** | |  | |  |  |
| *IL18BP* | 1.2 | -1.3 | |  | |  |  |
| *IL1RN* | -1.2 | -1.1 | |  | |  |  |
| *CX3CL1* | -1.4 | -1.2 | |  | |  |  |
| *CX3CR1* | -1.1 | 1.3 | |  | |  |  |
| *PDGFA* | -1.4 | 1.1 | |  | |  |  |
| *PDGFB* | 1.2 | -1.2 | |  | |  |  |
| *PDGFRA* | 1.6 | -1.4 | |  | |  |  |
| *PDGFRB* | 1.4 | -1.6 | |  | |  |  |
| *FGFR1* | 1.2 | 1.0 | |  | |  |  |
| *CNTFR* | -1.6 | 1.1 | |  | |  |  |
| *LIF* | 1.3 | 1.2 | |  | |  |  |
| *MANF* | -1.1 | -1.2 | |  | |  |  |
| *TGFB1* | 1.3 | 1.2 | |  | |  |  |
| *TGFB2* | 1.2 | 1.6 | |  | |  |  |
| *EGF* | 1.7 | 1.2 | |  | |  |  |
| *EGFR* | 1.1 | -1.2 | |  | |  |  |
| *IL4* | -1.6 | 1.2 | |  | |  |  |
| *IL13* | -1.1 | -1.2 | |  | |  |  |
| *NTRK2* | 1.6 | 1.5 | |  | |  |  |
| *NTRK3* | -1.4 | -1.1 | |  | |  |  |
| *IGF1* | -1.3 | 1.1 | |  | |  |  |
|  | ***5 wks*** | ***5 wks*** | |  | |  |  |
| *IL18BP* | 1.1 | -1.1 | |  | |  |  |
| *IL1RN* | 1.0 | 1.2 | |  | |  |  |
| *CX3CL1* | 1.2 | 1.3 | |  | |  |  |
| *CX3CR1* | 1.5 | 1.1 | |  | |  |  |
| *PDGFA* | -1.2 | 1.2 | |  | |  |  |
| *PDGFB* | -1.1 | 1.2 | |  | |  |  |
| *PDGFRA* | 1.5 | 1.7 | |  | |  |  |
| *PDGFRB* | 1.8 | 1.4 | |  | |  |  |
| *FGFR1* | 1.1 | 1.1 | |  | |  |  |
| *CNTFR* | -1.1 | 1.0 | |  | |  |  |
| *MANF* | 1.1 | -1.1 | |  | |  |  |
| *TGFB1* | 1.1 | 1.3 | |  | |  |  |
| *TGFB2* | -1.4 | 1.2 | |  | |  |  |
| *EGF* | 1.3 | 0.7 | |  | |  |  |
| *EGFR* | -1.4 | 1.1 | |  | |  |  |
| *IL4* | -1.6 | 1.2 | |  | |  |  |
| *IL13* | 1.1 | -1.1 | |  | |  |  |
| *NTRK2* | -1.2 | -1.3 | |  | |  |  |
| *NTRK3* | -1.6 | 1.2 | |  | |  |  |
| *IGF1* | -1.2 | 1.1 | |  | |  |  |
|  | ***7 wks*** | ***7 wks*** | |  | |  |  |
| *IL18BP* | 1.3 | 1.1 | |  | |  |  |
| *IL1RN* | -1.4 | -1.6 | |  | |  |  |
| *CX3CL1* | 1.5 | -1.4 | |  | |  |  |
| *PDGFA* | 1.3 | -1.1 | |  | |  |  |
| *PDGFB* | 1.8 | -1.4 | |  | |  |  |
| *PDGFRA* | 1.7 | -1.2 | |  | |  |  |
| *PDGFRB* | -1.4 | -1.6 | |  | |  |  |
| *FGFR1* | 1.2 | -1.4 | |  | |  |  |
| *CNTFR* | 1.1 | -1.4 | |  | |  |  |
| *MANF* | 1.2 | 1.1 | |  | |  |  |
| *TGFB1* | -1.2 | 1.2 | |  | |  |  |
| *TGFB2* | -1.4 | -1.1 | |  | |  |  |
| *IL13* | 1.3 | -1.1 | |  | |  |  |
| *NTRK2* | 1.1 | -1.2 | |  | |  |  |
| *NTRK3* | 1.8 | 1.1 | |  | |  |  |
| *IGF1* | -1.1 | 1.3 | |  | |  |  |
|  | ***16 wks*** | ***16 wks*** | | ***9.6-12 wks*** | | ***16 wks*** |  |
| *IL18BP* | 1.0 | 1.4 | | -1.1 | | 1.2 |  |
| *PDGFB* | 1.6 | -1.1 | | 1.1 | | 1.2 |  |
| *PDGFRA* | 1.6 | 1.2 | | -1.2 | | 1.1 |  |
| *CNTFR* | 1.5 | -1.1 | | 1.3 | | -1.2 |  |
| *MANF* | -1.2 | -1.1 | | 1.1 | | -1.3 |  |
| *TGFB2* | 1.2 | 1.1 | | -1.2 | | 1.1 |  |
| *IL13* | 1.4 | 1.1 | | -1.1 | | 1.2 |  |
| *NTRK2* | 1.3 | 1.2 | | 1.1 | | 1.3 |  |
| *NTRK3* | 1.7 | -1.6 | | 1.1 | | 1.2 |  |
| *IGF1* | -1.1 | 1.2 | | 1.4 | | 1.7 |  |
| *IL10* |  |  | |  | | 1.1 |  |
