# Supplementary material for: Strong upregulation of inflammatory genes accompanies photoreceptor demise in canine models of retinal degeneration
Source: PLoS One. 2017 May 9;12(5):e0177224. doi: 10.1371/journal.pone.0177224 (PMC5423635; doi:10.1371/journal.pone.0177224)
Supplement: S6 Table — Differentially expressed genes (p<0.05 and FC≥+/-2) are marked in red. (DOCX) [file pone.0177224.s008.docx]

**S6 Table. Comparative analysis of gene expression in study models: HDACs and HATs group.**

| **Genes** | **FC rcd1**  **vs. normal** | | **FC xlpra2**  **vs. normal** | **FC erd**  **vs. normal** | **FC xlpra1**  **vs. normal** | | |
| --- | --- | --- | --- | --- | --- | --- | --- |
|  | ***3 wks*** | ***3 wks*** | |  | |  |  |
| *HDAC1* | 1.3 | -1.1 | |  | |  |  |
| *HDAC2* | 1.0 | 1.2 | |  | |  |  |
| *HDAC3* | -1.3 | 1.1 | |  | |  |  |
| *HDAC4* | -1.4 | -1.4 | |  | |  |  |
| *HDAC5* | 1.1 | 1.3 | |  | |  |  |
| *HDAC6* | 1.4 | 1.3 | |  | |  |  |
| *HDAC9* | -1.1 | -1.4 | |  | |  |  |
| *SIRT1* | -1.5 | 1.0 | |  | |  |  |
| *SIRT2* | -1.1 | -1.3 | |  | |  |  |
| *KAT21* | 1.2 | 1.6 | |  | |  |  |
| *EP300* | 1.1 | 1.2 | |  | |  |  |
| *CREBBP* | 1.3 | -1.1 | |  | |  |  |
| *TAF1* | 1.1 | 1.4 | |  | |  |  |
|  | ***5 wks*** | ***5 wks*** | |  | |  |  |
| *HDAC1* | 1.3 | 1.4 | |  | |  |  |
| *HDAC2* | 1.1 | 1.2 | |  | |  |  |
| *HDAC3* | 1.2 | -1.1 | |  | |  |  |
| *HDAC4* | -1.3 | -1.2 | |  | |  |  |
| *HDAC5* | -1.4 | 1.1 | |  | |  |  |
| *HDAC6* | -1.1 | 1.4 | |  | |  |  |
| *HDAC9* | -1.1 | -1.2 | |  | |  |  |
| *SIRT1* | 1.2 | -1.3 | |  | |  |  |
| *SIRT2* | 1.1 | 1.2 | |  | |  |  |
| *KAT21* | 1.3 | 1.6 | |  | |  |  |
| *EP300* | 1.4 | 1.2 | |  | |  |  |
| *CREBBP* | 1.1 | 1.6 | |  | |  |  |
| *TAF1* | 1.3 | 1.1 | |  | |  |  |
|  | ***7 wks*** | ***7 wks*** | |  | |  |  |
| *HDAC1* | -1.1 | 1.2 | |  | |  |  |
| *HDAC2* | 0.7 | 1.1 | |  | |  |  |
| *HDAC3* | 1.1 | -1.2 | |  | |  |  |
| *HDAC4* | 1.3 | **-2.4** | |  | |  |  |
| *HDAC5* | -1.1 | **-2.0** | |  | |  |  |
| *HDAC6* | 1.2 | -1.4 | |  | |  |  |
| *HDAC9* | 1.3 | 1.0 | |  | |  |  |
| *SIRT1* | 1.4 | 1.1 | |  | |  |  |
| *SIRT2* | 1.1 | -1.1 | |  | |  |  |
| *KAT21* | 1.2 | 1.3 | |  | |  |  |
| *EP300* | 1.6 | 1.2 | |  | |  |  |
| *CREBBP* | 1.1 | -1.3 | |  | |  |  |
| *TAF1* | 0.9 | 1.2 | |  | |  |  |
|  | ***16 wks*** | ***16 wks*** | | ***9.6-12 wks*** | | ***16 wks*** |  |
| *HDAC1* | -1.2 | -1.1 | | -1.4 | | 1.3 |  |
| *HDAC2* | -1.1 | 1.3 | | 1.2 | | 1.1 |  |
| *HDAC3* | 1.1 | 1.2 | | 1.0 | | -1.2 |  |
| *HDAC4* | 1.4 | 1.1 | | -1.5 | | 1.4 |  |
| *HDAC5* | -1.2 | 1.2 | | -1.2 | | 1.1 |  |
| *HDAC6* | 1.1 | -1.3 | | 1.1 | | 1.1 |  |
| *HDAC9* | -1.4 | -1.2 | | **-2.3** | | -1.3 |  |
| *SIRT1* | 1.3 | 1.1. | | -1.2 | | -1.1 |  |
| *SIRT2* | 1.2 | 1.3 | | 1.1 | | 1.2 |  |
| *KAT21* | 1.6 | 1.2 | | 1.3 | | -1.7 |  |
| *EP300* | 1.1 | -1.1 | | -1.1 | | **-2.0** |  |
| *CREBBP* | 1.2 | -1.3 | | 1.4 | | 1.1 |  |
| *TAF1* | 1.4 | 1.2 | | 1.1 | | -1.4 |  |

Differentially expressed genes (p<0.05 and FC≥+/-2) are marked in red.
